# Cyclin L1 controls cardiomyocyte proliferation and heart repair after injury

**DOI:** 10.1038/s41392-023-01444-1

**Published:** 2023-06-21

**Authors:** Rui Gong, Xinlu Gao, Yu Liu, Yifu Shen, Zuke Jiang, Xiuxiu Wang, Naufal Zagidullin, Wenya Ma, Ning Wang, Benzhi Cai

**Affiliations:** 1 Department of Pharmacy at The Second Affiliated Hospital, and Department of Pharmacology at College of Pharmacy (The Key Laboratory of Cardiovascular Medicine Research, Ministry of Education), Harbin Medical University, 150081 Harbin, China; 2 Department of Laboratory Medicine at The Fourth Affiliated Hospital, Harbin Medical University, 150086 Harbin, China; 3 Department of Internal Diseases, Bashkir State Medical University, Ufa, 450008 Russia

**Keywords:** Cardiovascular diseases, Gene therapy, Cardiology

**Dear Editor**,

Myocardial infarction (MI) is characterized by the loss of functional cardiomyocyte (CM) in the heart, resulting in cardiac systolic dysfunction and heart failure.^1,2^ Increasing evidence suggested that in the heart of neonatal mice after apical resection (AR), the CM can proliferate and regenerate myocardium to repair the heart. While in the heart of adult mice after MI, the CM loses the ability to re-enter the cell cycle but undergoes hypertrophic growth, which contributes to cardiac pathological remodeling.^3,4^ Thus, inducing adult CM cell cycle re-entry is a novel strategy to promote the repair of damaged hearts and improve cardiac function. It has been reported that the cyclin family plays an important role in cell cycle regulation.^5^ However, it was not known whether Cyclin L1 (CCNL1), a member of the cyclin family, regulates CM proliferation and heart repair after injury.

Here, we performed immunofluorescence staining on CM and found that CCNL1 is mainly expressed in the nucleus (Supplementary Fig. 1a), and its expression was markedly upregulated in the mouse heart tissue after birth (Fig. 1a). While 7 days after AR in 1-day-old mice, the expression of CCNL1 was significantly decreased in heart tissue of mice (Supplementary Fig. 1b, c). Additionally, CCNL1 expression was markedly enhanced in heart tissue of mice with MI (Supplementary Fig. 1d). These data strongly implies that CCNL1 may be involved in the regulation of cardiomyocyte proliferative potential after injury. To investigate the regulatory effect of CCNL1 on CM proliferation, we performed immunofluorescence staining and found that CCNL1 negatively regulates CM proliferation in vitro (Fig. 1b and Supplementary Fig. 1e–i). The similar results were obtained in HL-1 cell line as well (Supplementary Fig. 2). We next constructed cTnT-CCNL1 shRNA adeno-associated virus 9 (AAV-9 CCNL1 shRNA) to explore whether CCNL1 silencing could promote heart repair after injury. As expected, after AAV-9 CCNL1 shRNA was administered to MI mice, the pre-existing CM re-entered the cell cycle, the CM size and infarct size were decreased, and the damaged cardiac function was significantly improved (Fig. 1c, d and Supplementary Fig. 3). The above data indicate that the silencing of CCNL1 promotes CM proliferation and heart repair after MI.

**Fig. 1:**
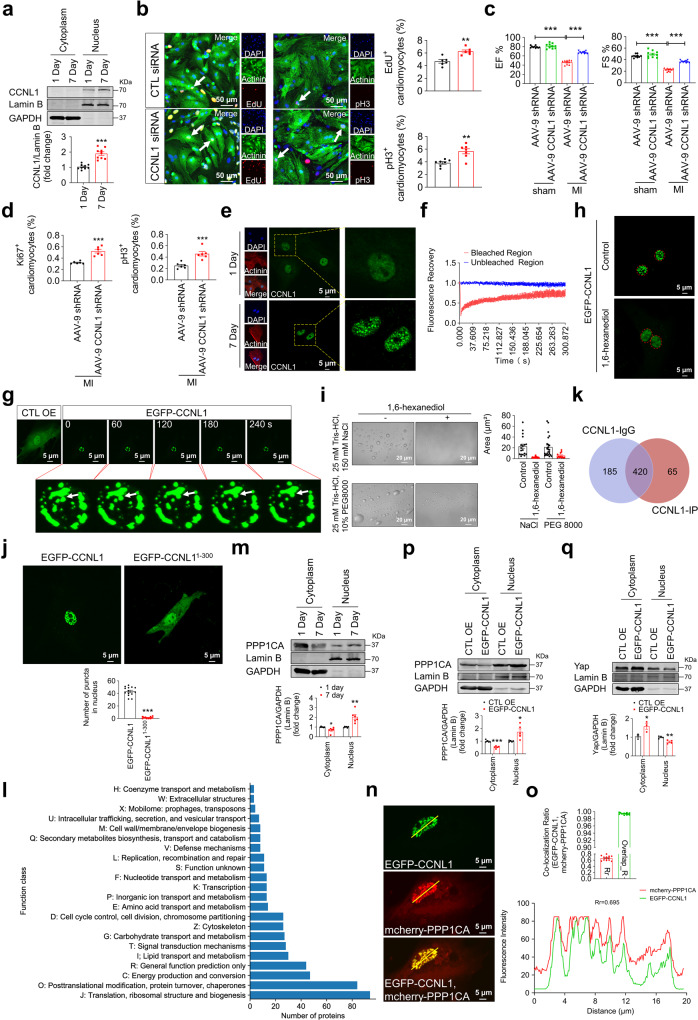
CCNL1 plays a regulatory role in CM proliferation and heart repair after MI, which is partially related to its LLPS behavior. **a** Western blot analysis of CCNL1 expression and localization in the nucleus and cytoplasm of the heart tissues from 1-day-old and 7-day-old mice. Lamin B was served as a loading control for the nucleus and GAPDH for the cytoplasm. Data were represented as means ± SEM (*n* = 9; ***P < 0.001). **b** The neonatal CM was transfected with small interfering RNA-targeting CCNL1 (CCNL1 siRNA) and corresponding control siRNA (CTL siRNA), and the CM proliferation was evaluated by immunofluorescence staining of EdU, pH3 and Actinin (marked CM). The arrows point to EdU/pH3-positive signal in CM. Data were represented as means ± SEM (EdU: *n* = 6; pH3: *n* = 7; **P < 0.01). Scale bar: 50 µm. **c** The cardiac function of adult MI mice 28 days after administration of AAV-9 CCNL1 shRNA was analyzed by echocardiography. ANOVA test was used (*n* = 10; ***P < 0.001). **d** Quantification of the percentage of Ki67/pH3^+^ CM in the heart tissue of MI mice 28 days after administration of AAV-9 CCNL1 shRNA. Data were represented as means ± SEM (*n* = 6; ***P < 0.001). **e** The expression and localization of CCNL1 in the CM of 1-and 7-day-old mice were analyzed by immunofluorescence staining. Scale bars: 5 µm. **f** The CM was transfected with EGFP-CCNL1 for 48 h and FRAP was performed. Quantitative FRAP data are shown as mean ± SEM. A typical FRAP recovery curve of puncta of EGFP-CCNL1 averaged from *n* = 6 biological replicates. **g** Time-lapse imaging of the fusion behavior of puncta of EGFP-CCNL1 in the nucleus of CM. The time interval is 60 s. *n* = 4 biological replicates. Scale bars: 5 µm. **h** The effect of 1,6-hexanediol (5%) treatment on the formation of puncta in neonatal CM transfected with exogenous EGFP-CCNL1. The red dotted circle indicates the nucleus. Scale bars: 5 µm. **i** The effect of 1,6-hexanediol on droplet formation of CCNL1 (with IDRs) protein purified in vitro in the presence of salt solution (150 mM NaCl) or crowding agent (10% PEG 8000). Representative differential interference contrast (DIC) images of the droplets and quantification of the size of droplets are shown. Each dot represents a field (8.67 × 8.67 cm^2^). *n* = 3 biological replicates. Scale bars: 20 µm. **j** The effect of the IDRs of CCNL1 on the formation of puncta in neonatal CM. After transfection of EGFP-CCNL1 and EGFP-CCNL1^1–300^ plasmid in neonatal CM, the images of puncta formation were collected by Live-cell imaging. The number of puncta is quantified. Each dot represents the number of puncta in the CM nucleus under a field. Data were represented as means ± SEM (*n* = 14; ***P < 0.001). Scale bars: 5 µm. **k** Venn diagram indicates the overlapping and unique proteins identified from the two groups (CCNL1-IgG and CCNL1-IP) by Co-IP/LC-MS analysis. **l** Cluster of Orthologous Groups (COG) analysis of proteins using Co-IP/LC-MS analysis data. PPP1CA is enriched in the category of “Signal Transduction mechanisms”. **m** Western blot analysis of PPP1CA expression and localization in the nucleus and cytoplasm of the heart tissues from 1-and 7-day-old mice. Lamin B was served as a loading control for the nucleus and GAPDH for the cytoplasm. Data were represented as means ± SEM (*n* = 6; *P < 0.05, **P < 0.01). **n**, **o** EGFP-CCNL1 plasmid and PPP1CA-overexpressing plasmid (mcherry-PPP1CA) were transfected into neonatal CM, and the co-localization of EGFP-CCNL1 (green) and mcherry-PPP1CA (red) was observed by confocal microscopy. The fluorescence intensity trace (yellow line) is shown on the right. The quantification of the Rr and Overlap_R is shown in bar graph format (13 cells). Scale bar: 5 µm. Rr: Pearson’s_Rr, indicates Pearson’s correlation; Overlap_R: overlap coefficient. **p**, **q** Western blot analysis of PPP1CA and Yap expression and localization in the nucleus and cytoplasm of CM transfected with EGFP-CCNL1. Lamin B was served as a loading control for the nucleus and GAPDH for the cytoplasm. Data were represented as means ± SEM (*n* = 3–5; *P < 0.05, **P < 0.01, ***P < 0.001)

Furthermore, we performed immunofluorescence staining of CCNL1 on the CM isolated from postnatal mice, and found that CCNL1 in the CM nucleus of 7-day-old mice exhibited larger “puncta” than that of 1-day-old mice (Fig. 1e). Meanwhile, we found that the full length CCNL1 protein contains many intrinsically disordered regions (IDRs) (Supplementary Fig. 4). Given that proteins with IDRs that may participate in the interaction of multivalent proteins tend to undergo liquid–liquid phase separation (LLPS),^6^ we speculated that CCNL1 may undergo LLPS in the CM of postnatal mice. It was reported that sphericity, ability to split and fuse, and rapid and spontaneous fluorescence recovery after photobleaching (FRAP) are key features of phase-separated condensates.^7^ Thus, we conducted FRAP analysis and found that the puncta of EGFP-CCNL1 in the nucleus of CM and HL-1 cell line exhibited rapid fluorescence recovery after photobleaching (Fig. 1f and Supplementary Fig. 5a, b). Live-cell imaging showed that the nuclear puncta of EGFP-CCNL1 would gradually fuse and become larger (Fig. 1g and Supplementary Fig. 5c). These results suggest that the puncta of EGFP-CCNL1 in the CM nucleus is dynamic and exhibit liquid properties. In addition, the puncta of EGFP-CCNL1 in the CM nucleus was destroyed after treatment with 1,6-hexanediol (which disrupted droplet aggregation by disrupting hydrophobic interactions) (Fig. 1h). The purified CCNL1 (containing IDRs) protein formed droplets spontaneously in salt solutions and in solutions containing crowding agent, and most droplet formation was inhibited by treatment with 1,6-hexanediol (Fig. 1i). The above data suggest that CCNL1 has the ability to phase separate into condensate in the CM. Then, we tested the effect of CCNL1 undergoing LLPS on CM proliferation. Live-cell imaging showed that the EGFP-CCNL1 without IDRs (EGFP-CCNL1^1–300^) was expressed in the whole CM, and no obvious puncta similar to EGFP-CCNL1 was observed in the CM nucleus (Fig. 1j). Notably, transfection with EGFP-CCNL1^1–300^ did not inhibit CM proliferation compared with EGFP-CCNL1 (Supplementary Fig. 6a). Meanwhile, the proliferation ability of CM was enhanced after LLPS of CCNL1 was destroyed by 1,6-hexanediol (Supplementary Fig. 6b). These results indicate that the inhibitory effect of CCNL1 on CM proliferation is at least partially associated with its LLPS behavior.

To further elucidate how CCNL1 affects CM proliferation, the proteins interacting with CCNL1 were determined by Co-immunoprecipitation/liquid chromatography mass spectrometry (Co-IP/LC-MS). A total of 65 proteins were identified in the immunoprecipitated CCNL1 complex and the Co-IP/LC-MS analysis data were classified according to biological function (Fig. 1k, l). Cluster of Orthologous Groups (COG) analysis and protein enrichment analysis suggested that the proteins are highly enriched in the category of “signal transduction” (Supplementary Fig. 7). The above data showed that CCNL1 may play a crucial role in regulating CM proliferation through intracellular signal transduction. Among the candidate proteins immunoprecipitated by CCNL1 in the category of “signal transduction mechanisms” (Fig. 1l), PPP1CA, a protein phosphatase involved in the dephosphorylation of key factors of Hippo signaling pathway, such as yes-associated protein (Yap), played a role in regulating CM proliferation.^8,9^ The expression of PPP1CA was obviously elevated in the nucleus of mouse heart tissue after birth, which was consistent with CCNL1 (Fig. 1m). To test whether CCNL1 could interact with PPP1CA, we performed Live-cell imaging and Co-IP and found that CCNL1 does interact with PPP1CA (Fig. 1n, o and Supplementary Fig. 8a). Furthermore, we found that overexpression of CCNL1 significantly increases the nuclear expression of PPP1CA and decreases the cytoplasm expression of PPP1CA (Fig. 1p). Our previous study has found that PPP1CA silencing inhibits the dephosphorylation of phosphorylated Yap and reduces Yap nuclear translocation, thereby depressing CM proliferation.^9^ Thus, we further analyzed the effect of CCNL1 on the expression of Yap in CM. The results showed that the nuclear translocation of Yap was decreased after CCNL1 overexpression, while CCNL1 siRNA promoted Yap nuclear translocation, which was partially blocked by PPP1CA siRNA (Fig. 1q and Supplementary Fig. 8b). Moreover, we found that knockdown of PPP1CA and Yap partially attenuates the effects of CCNL1 silencing and PPP1CA overexpression on CM proliferation, respectively (Supplementary Fig. 8c–e).

The above results suggest that elevated concentration of CCNL1 can undergo LLPS in the CM nucleus of postnatal mice and inhibit CM proliferation, which is at least partially associated with CCNL1/PPP1CA nuclear accumulation and the less nuclear translocation of Yap (Supplementary Fig. 8f). Of course, the connection between the regulatory effect of CCNL1 on CM proliferation and heart repair after injury and its LLPS behavior still needs to be further explored. Additionally, it is reported that CDK/cyclin-dependent phosphorylation control alternative splicing, such as CDK11/cyclin L complexes.^10^ So, the CDK11/CCNL complexes might also be involved in the regulation of CM proliferation and heart repair after injury by affecting alternative splicing of mRNAs. Taken together, we confirmed that CCNL1 plays a regulatory role in CM proliferation and heart repair after MI, which may be a potential regulatory target for heart repair after MI.

## Supplementary information

Supplementary Materials

## Data Availability

All data and materials are presented in the main manuscript or supplementary materials, and can be obtained from the corresponding authors upon authorization or reasonable request.
