# Supplementary material for: Cyclin L1 controls cardiomyocyte proliferation and heart repair after injury
Source: Signal Transduct Target Ther. 2023 Jun 21;8:243. doi: 10.1038/s41392-023-01444-1 (PMC10282026; doi:10.1038/s41392-023-01444-1)
Supplement: Supplementary file 1 — Supplementary Materials [file 41392_2023_1444_MOESM1_ESM.docx]

Supplementary Materials for

**Cyclin L1 controls cardiomyocyte proliferation and heart repair after injury**

Rui Gong^1^*, Xinlu Gao^1^*, Yu Liu^2^*, Yifu Shen^2^*, Zuke Jiang^1^, Xiuxiu Wang^1^, Naufal Zagidullin^3^, Wenya Ma^1^, Ning Wang^1^, Benzhi Cai^1^

**Author affiliations and contact information**

^1^ Department of Pharmacy at The Second Affiliated Hospital, and Department of Pharmacology at College of Pharmacy (The Key Laboratory of Cardiovascular Medicine Research, Ministry of Education), Harbin Medical University, Harbin 150081, China

^2^ Department of Laboratory Medicine at The Fourth Affiliated Hospital, Harbin Medical University, Harbin 150086, China

^3^ Department of Internal Diseases, Bashkir State Medical University, Ufa, 450008, Russia

*These authors made equivalent contributions in this study.

Correspondence to: [caibz@ems.hrbmu.edu.cn](mailto:caibz@ems.hrbmu.edu.cn)

**This PDF file includes:**

Materials and Methods

Figures. S1 to S8

Table S1

Original result of Co-IP/LC-MS

Original films of Western Blot

Materials and Methods

**Mice**

The neonatal (1-3 days old) mice or adult (6-8 weeks old) male C57BL/6 mice were purchased from the Experimental Animal Center of The Second Affiliated Hospital of Harbin Medical University (Harbin, China). All animal experiments were performed according to the EU (Directive 2010/63/EU) ethical guidelines and were approved by the Institutional Animal Care and Use Committee of Harbin Medical University. Mice were housed at SPF level and anesthetized with isoflurane (3%, inhalation) by continuous anesthesia device, and euthanized by cervical dislocation (CD).

**Isolation and culture of neonatal CM**

In this study, the neonatal CM was obtained by enzymatic digestion. In brief, the mouse heart was first dissected at 1 (P1) or 7 days (P7) after birth and divided into small pieces. Then, the heart pieces were subjected to multiple rounds of digestion separation with trypsin EDTA solution (Solarbio, China) after washing three times with phosphate buffer saline (PBS). And the cell digests obtained each time were resuspended with Dulbecco's modified eagle's medium (DMEM) containing 10% fetal bovine serum (FBS) and 1% penicillin-streptomycin. Finally, the cell suspension was centrifuged at 500g for 7 min to obtain a monolayer of neonatal CM, which was resuspended with DMEM containing 10% FBS and 1% penicillin-streptomycin, and inoculated on a culture plate coated with 0.1% gelatin (g1890, sigma) and cultured at 37°C and 5% CO_2_.

**Transfection of small interfering RNA and plasmid**

The plasmids were transfected into neonatal CM using the transfection reagent Lipofectamine 2000 (Invitrogen, USA) and the siRNAs were transfected into neonatal CM by Lipofectamine RNAiMAX (Invitrogen, USA), as described in the manufacturer's protocol. The CCNL1-overexpressing plasmid (EGFP-CCNL1), EGFP-CCNL1^1-300^ plasmid, and PPP1CA-overexpressing plasmid (mcherry-PPP1CA) were purchased from GENERAL BIOSYSTEMS (An Hui, China), and CCNL1 siRNA, PPP1CA siRNA, Yap siRNA and negative control siRNA (CTL siRNA) were purchased from GenePharma (Shanghai, China). The neonatal CM can be subjected to subsequent immunofluorescence staining and Western blot analysis 48 h after transfection. The corresponding sequences are shown in Supplementary Table 1.

**Live-cell imaging of neonatal CM**

The neonatal CM was transfected with EGFP-CCNL1 plasmid or EGFP-CCNL1^1-300^ plasmid or co-transfected with EGFP-CCNL1 plasmid and mcherry-PPP1CA plasmid and scanned under a confocal laser scanning microscope (FV10i; Olympus, Japan). The time-lapse images of EGFP-CCNL1 are taken continuously at a time interval of 60 s with the same environment and parameters. 1, 6-hexanediol was used in this experiment at a concentration of 5%. For the co-localization of CCNL1 and PPP1CA in neonatal CM, Image J software (NIH) was used to fit the "fluorescence intensity-distance" curve between EGFP-CCNL1 and mcherry-PPP1CA. The Pearson’s correlation, overlap coefficient and the number of puncta of EGFP-CCNL1 and EGFP-CCNL1^1-300^ were statistically analyzed.

**Fluorescence recovery after photobleaching (FRAP)**

FRAP was performed using a Zeiss LSM 800 confocal microscope with a 488 nm laser. Briefly, FRAP experiments were conducted on neonatal CM with Zen bleaching mode to investigate the fluorescence recovery of puncta of EGFP-CCNL1 after photobleaching. A suitable sized area of interest was selected in the nucleus of CM and bleached 100 times with a 488 nm laser at 100% power. The images were scanned 3 times and about 300 times before and after bleaching until the fluorescence signal was stable. The "fluorescence recovery-time" curves of EGFP-CCNL1 before and after bleaching were fitted using Prism 8.0 software.

**In vitro pSMARt-I-CCNL1 expression, protein purification and droplet formation assay**

The pSMARt-I-CCNL1 plasmid was transformed into Escherichia coli BL21(DE3) cells according to standard supplier protocols. The mono-clones on the transformation plate were selected and grown to an optical density (OD600) of 0.6-0.8. The isopropyl-β-d-thiogalactoside (IPTG) (Sigma) was added to the culture to a final concentration of 0.4 mM and shaken overnight at 15℃ to induce the expression of fusion protein. After ultrasonic fracturing, the fusion proteins were purified by Ni-IDA-Sepharose Cl-6B affinity chromatography. The eluted proteins were dialyzed overnight with 20 mM Tris-HCl, 0.15 M NaCl, pH 8.0 and concentrated with an ultrafiltration Centrifugal Filters (Millipore). The purified protein was concentrated to approximately 3.3 mg ml^-1^, 20 mM Tris, pH 8.0.

For droplet formation assay in vitro, the purified CCNL1 protein was diluted at a concentration of 20 μM in buffer containing 25 mM Tris-HCl with 150 mM NaCl or crowding agent (10% PEG 8000). The protein buffer was loaded onto cell culture dish (801002, NEST) and the images were scanned with Zeiss LSM 800 confocal microscope. The fields were quantified using Image J software (NIH).

**Co-Immunoprecipitation/liquid chromatograph-mass spectrometer (Co-IP/LC-MS)**

The neonatal CM or CM transfected with EGFP-CCNL1 plasmid were fully lysed with non-denatured cell/tissue lysis buffer (Solarbio, China) and their concentration was assessed by the BCA Protein Assay Kit (Beyotime, China) for quantification. 1-2 mg protein was incubated with anti-CCNL1 antibody (Cat No. GTX48663; GeneTex) or control IgG (cat. no-3900 s, Cell Signaling) on a vertical shaker for overnight at 4°C. The “antigen-antibody” mixture was added to the protein A/G magnetic beads washed three times with 0.2% PBS-Tween (PBST), and incubated on a vertical shaker for overnight at 4°C. After elution, the samples were mixed with SDS-PAGE protein loading buffer (Beyotime, China) and subjected to Western blot analysis. The gel bands were digested for LC/MS analysis. The database used is Mus_musculus_uniprot_2021_7_15. fasta. Fasta (86544 sequences) and the databases of Clusters of Orthologous Groups (COG) was used to analyze the protein family. The antibodies used in the experiments were as follows: anti-PPP1CA (1:300; ab150782, Abcam) and anti-CCNL1 (1:200; Cat No. GTX48663, GeneTex). The groups are Input, IgG and IP. IgG was used as negative control.

**Apical resection (AR)**

The 1-day-old mice were anesthetized by cooling on the ice bed for 3-5 min. After their skin was sterilized, the skin was cut with iris excision scissors, and then the muscles were bluntly separated with surgical forceps to open the chest cavity. The apical part of the heart was immediately removed with iris excision scissors quickly and accurately. The chest was closed immediately after cleaning out the blood and placed it in a warm environment until the mice woke up. In sham group, the mice underwent the same procedure, but did not remove the apical part of the heart.

**Myocardial infarction (MI) model and administration of** **cTnT-CCNL1 shRNA adeno-associated virus 9 (AAV-9 CCNL1 shRNA)**

Adult mice aged 6 weeks were randomly selected to establish the acute MI model. Then, 4×10^10^ AAV-9 CCNL1 shRNA and relevant control, purchased from Hanbio (Shanghai, China), were delivered to the infarcted heart at an injection volume of 50 μl/mice. In general, after anesthetizing mice with isoflurane, we performed blunt thoracotomy and permanently ligated the left anterior descending artery (LAD) with 7-0 prolene sutures, and injected AAV-9 CCNL1 shRNA into the heart. Before waking up, the mouse's chest was closed. 28 days after surgery, the heart samples were collected for subsequent analysis and evaluation.

**Echocardiography**

The measurement and evaluation of cardiac function were performed by using Vevo 1100 VisualSonics device (VisualSonics, Toronto, ON, Canada). Specifically, we obtained the M-mode image by scanning the long-axis projection of the heart after completing the cardiac cavity imaging, and evaluated the left ventricular ejection fraction (EF%) and shortening fraction (FS%) of the mice through VisualSonics V1.3.8 software. All the echocardiographic measurements are random and objective.

**Ethynyl-2-deoxyuridine (EdU)** **incorporation**

DNA synthesis was evaluated by incorporation of 5-ethynyl-2`-deoxyuridine into transfected CM using the Apollo 567 in Vitro Kit (Ribobio, Guangzhou, China) according to the manufacturer's instructions. Briefly, when the neonatal CM was transfected with EGFP-CCNL1 plasmid, EGFP-CCNL1^1-300^ plasmid or CCNL1 siRNA for 36 h, 5-ethynyl-2`-deoxyuridine was added to the cells and incubated for 12 h to complete the incorporation. After that, the CM incorporated with 5-ethynyl-2`-deoxyuridine was labeled by Apollo mixture reagent, and the pictures were collected by a laser confocal microscope (FV10i; Olympus, Japan) and the ratio was counted to evaluate the DNA synthesis.

**Immunofluorescence**

The CM treated with transfection or 1, 6-hexanediol and isolated from the hearts of P1 and P7 mice was incubated with the primary antibodies for overnight at 4°C and with the secondary antibody at room temperature (RT) for 1 h after being treated with fixation (4% paraformaldehyde (PFA), 15 min, 37°C), penetration (penetration buffer: PBS containing 0.4% Triton X-100, 45 min, RT), and blocking (blocking buffer: goat serum, 1 h, 37°C). The CM was counter-stained with DAPI to label the nuclei. The images were captured under a confocal laser scanning microscope (FV10i; Olympus, Japan). The following antibodies were used: anti-sarcomeric alpha Actinin antibody (EA-53) (1:400; GTX29465, GeneTex), anti-CCNL1 (1:200; AF0236, Affinity), anti-phospho histone H3 (Ser10) (1:400; #06-570, Millipore, Billerica, MA, USA), anti-Aurora B (1:400; ab2254, Abcam), goat anti-mouse IgG (H&L) (Alexa Fluor 488; 1:400; ab150113/ab150077, Abcam) and goat anti-rabbit IgG (H&L) (Alexa Fluor 594; 1:400; ab150080, Abcam).

**RNA extraction and** **Real-time quantitative PCR (qRT-PCR)**

Total RNA was extracted from CM transfected with EGFP-CCNL1 or CCNL1 siRNA by using Trizol. Purity and concentration of RNA were measured by Nanodrop 2000. Using High-Capacity cDNA Reserve Transcription Kit and gene amplification instrument, the RNA samples can be reverse transcribed into cDNA samples according to its instructions. Real-time quantitative PCR was performed with SYBR Green Realtime PCR Master Mix and ABI 7500 Fast sequence detection system. The sequence of primer for CCNL1 is as follows: Forward primer: 5'-ACCGCCTGTATTCGGAGGT-3', Reverse primer: 5'-GACTTGGAGTAGAAGAACCGATG-3'.

**Western blot**

The cytoplasmic and nuclear proteins were extracted from the heart of P1 and P7 mice, the heart tissues subjected to AR, and the CM transfected with EGFP-CCNL1 plasmid for 48 h by using Minute™ Cytosolic and Nuclear Extraction Kit for Frozen/Fresh Tissues (Invent biotechnologies, Inc; NT-032) and Minute™ Cytoplasmic & Nuclear Extraction Kits for Cells (Invent biotechnologies, Inc; SC-003) according to the manufacturer's instructions, and the protein concentration was evaluated by using the BCA Protein Assay Kit (Beyotime, China). The samples were loaded onto 10% SDS-PAGE gel. After separation, the proteins were transferred onto nitrocellulose membranes (Millipore, Bedford, MA, USA) and blocked with 5% non-fat milk in PBS for 1-2 h at RT. Finally, the membranes were incubated with primary antibodies for overnight at 4°C. Next day, the images were revealed through the binding of the secondary antibodies and primary antibodies and scanned with Odyssey (LI-COR Biosciences, Lincoln, NE, USA). The major antibodies used are as follows: anti-CCNL1 (1:1000; AF0236, Affinity), anti-PPP1CA (1:2000; 67070-1-Ig, Proteintech), anti-Yap (1:1000; 13584-1-AP, Proteintech), anti-Lamin B (1:1000; A1910, Abclonal) and anti-GAPDH (1:1000; AC033, Abclonal).

**Immunohistochemistry**

After 28 days of administration of AAV-9 CCNL1 shRNA in adult MI mice, the heart tissues were collected and cut into 5 μm sections using a freezing microtome. Next, after fixing with acetone (15 min, RT), permeating with 3% H_2_O_2_ (30 min, RT), and blocking with goat serum (1 h, 37°C), the heart sections were incubated with primary antibody for overnight at 4°C and with the secondary antibody for 1 h at RT, and finally counter-stained with DAPI to label nuclei. To quantify CM size, the fixed heart sections were stained with wheat germ agglutinin (WGA) conjugated with Alexa 488 (1:400; W11261, Thermo Fisher) at RT in the dark for 20 min. The images were scanned with a fluorescent microscope (Bx50, Olympus, Japan) and a laser confocal microscope (FV10i; Olympus, Japan) and the ratio of Ki67^+^, pH3^+^ or Aurora B^+^ CM was counted by using Image J software (NIH). The antibodies involved are listed as follows: anti-sarcomeric alpha Actinin antibody (EA-53) (1:400; GTX29465, GeneTex), anti-Ki67 antibody (1:400; ab15580, Abcam), anti-phospho histone H3 (Ser10) (1:400; #06-570, Millipore, Billerica, MA, USA), anti-Aurora B (1:400; ab2254, Abcam), goat anti-mouse IgG (H&L) (Alexa Fluor 488; 1:400; ab150113, Abcam) and goat anti-rabbit IgG (H&L) (Alexa Fluor 594; 1:400; ab150080, Abcam).

**Histology**

After 28 days of administration of AAV-9 CCNL1 shRNA in adult MI mice, the heart tissues were collected and cut into 6 μm sections and fixed with 4% PFA. The heart sections were then treated according to the following standard procedure: dehydration (Xylene, 10 min→Xylene, 10 min→100% ethanol, 7 min→100% ethanol, 7 min→95% ethanol, 7 min→95% ethanol, 7 min→90% ethanol, 7 min→85% ethanol, 7 min→80% ethanol, 7 min→75% ethanol, 7 min→water, 10 min→ddH_2_O, 10 min). Then, according to standard procedures, the Masson trichrome staining (Solarbio, China) was used to evaluate the infarct size.

**Statistics**

Statistical analysis was performed using Prism 8.0 software. Data were represented as means ± SEM. An Unpaired (two-tailed) student's t test was used to analyze the two groups of data. Multiple groups of data were analyzed using one-way analysis of variance (ANOVA) followed by Tukey’s post-hoc analysis. **P* < 0.05, ***P* < 0.01, ****P* < 0.001. The difference was considered statistically significant when the *P* value ≤0.05.

**Supplementary figures**

**
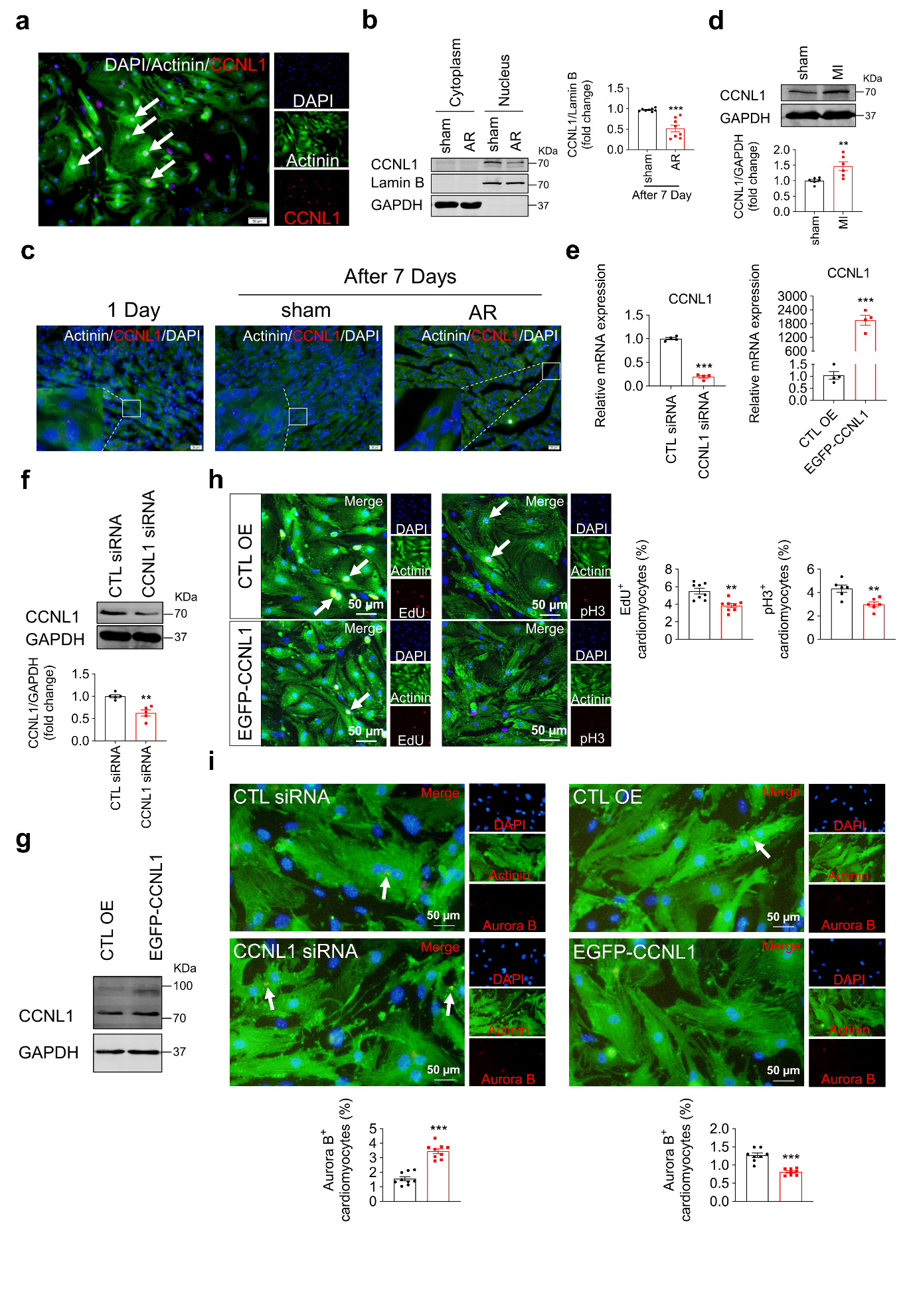
**

**Figure S1. The expression and localization and the overexpression and knockdown efficiency of CCNL1 in neonatal CM.** **a** Evaluation of CCNL1 intracellular localization in neonatal CM. Actinin marked CM. DAPI co-stains the nucleus. Scale bar: 50 µm. **b** Western blot analysis of CCNL1 expression and localization in nucleus and cytoplasm of the heart tissues from 7-day-old mice and 1-day-old mice 7 days after AR. Lamin B was served as a loading control for the nucleus and GAPDH for the cytoplasm. Data were represented as means ± SEM (n = 8; ****P* < 0.001). **c** Immunofluorescence staining was used to evaluate the expression and localization of CCNL1 in the heart tissue of 1-day-old and 7-day-old mice and 1-day-old mice after 7 days of AR. DAPI co-stains the nucleus. Scale bar: 20 µm. **d** Western blot analysis of CCNL1 expression in the heart tissues of sham and MI mice. GAPDH was served as a loading control in Western blot. Data were represented as means ± SEM (n = 6; ***P* < 0.01). **e** qRT-PCR analysis of the transfection efficiency of CCNL1 small interfering RNA-targeting CCNL1 (CCNL1 siRNA) or CCNL1-overexpressing plasmid (EGFP-CCNL1). Data were represented as means ± SEM (n = 4; ****P* < 0.001). **f** Western blot analysis of transfection efficiency of CCNL1 siRNA in CM. GAPDH was served as a loading control in Western blot. Data were represented as means ± SEM (n = 5; ***P* < 0.01). **g** Western blot analysis of transfection efficiency of EGFP-CCNL1 in CM. GAPDH was served as a loading control in Western blot. **h** The neonatal CM was transfected with CTL plasmid (CTL OE) and EGFP-CCNL1, and the CM proliferation was evaluated by immunofluorescence staining of EdU, pH3 and Actinin (marked CM). The arrows point to EdU/pH3-positive signal in CM. Data were represented as means ± SEM (EdU: n = 8; pH3: n = 6; ***P* < 0.01). Scale bar: 50 µm. **i** The neonatal CM was transfected with CCNL1 siRNA and EGFP-CCNL1, and the CM proliferation was evaluated by immunofluorescence staining of Aurora B and Actinin (marked CM). The arrows point to Aurora B-positive signal in CM. Data were represented as means ± SEM (n = 8-9; ****P* < 0.001). Scale bar: 50 µm.


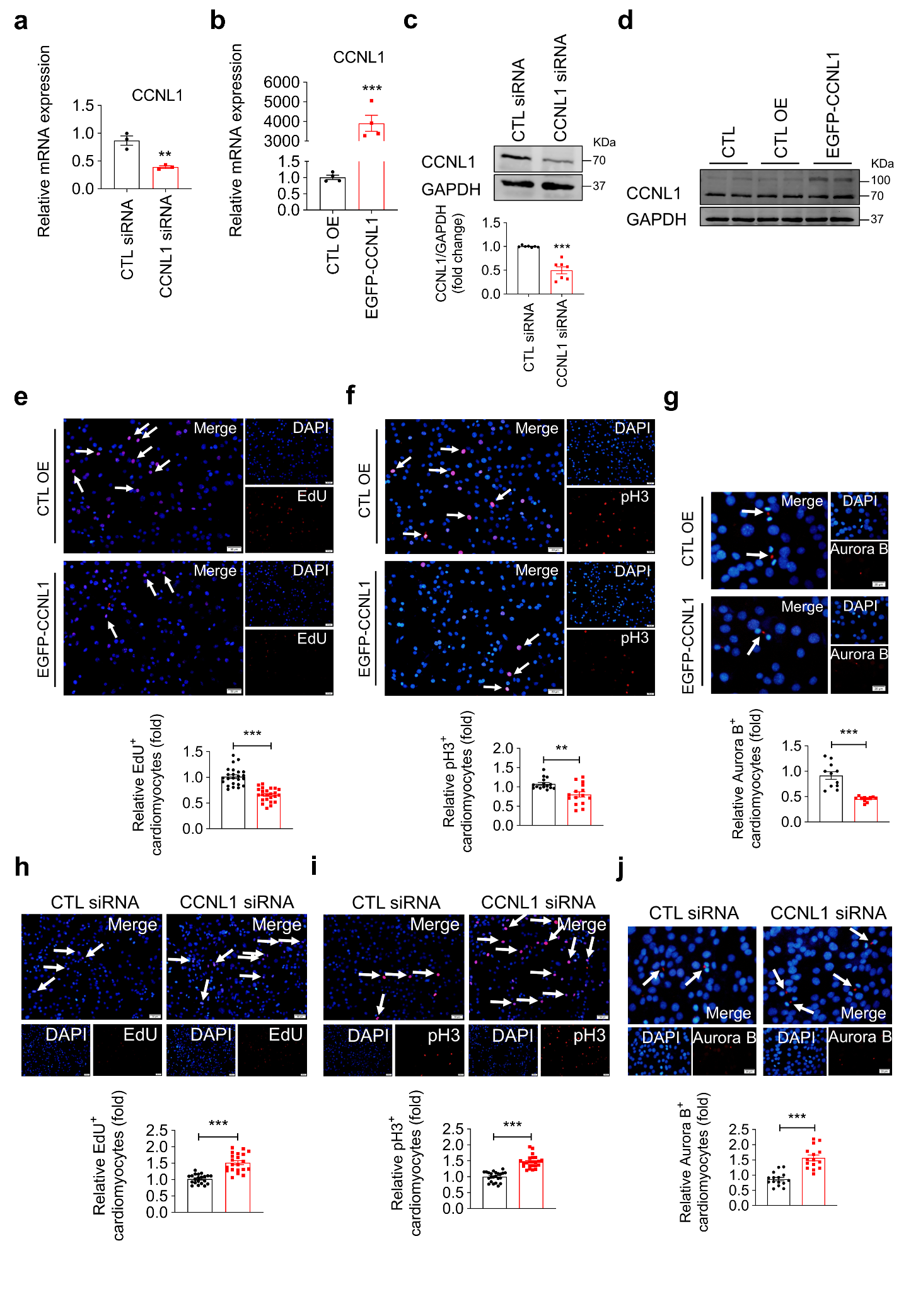


**Figure S2. CCNL1 negatively regulates the proliferation of HL-1** **cell line. a** qRT-PCR analysis of transfection efficiency of CCNL1 siRNA. Data were represented as means ± SEM (n = 3; ***P* < 0.01). **b** qRT-PCR analysis of transfection efficiency of EGFP-CCNL1 plasmids. Data were represented as means ± SEM (n = 4; ****P* < 0.001). **c** Western blot analysis of transfection efficiency of CCNL1 siRNA in HL-1 cell line. GAPDH was served as a loading control in Western blot. Data were represented as means ± SEM (n = 7; ****P* < 0.001). **d** Western blot analysis of transfection efficiency of EGFP-CCNL1 plasmids in HL-1 cell line. GAPDH was served as a loading control in Western blot. **e-g** HL-1 cell line was transfected with CTL plasmids (CTL OE) and EGFP-CCNL1 plasmids, and the CM proliferation was evaluated by immunofluorescence staining with EdU, pH3 and Aurora B. The arrows point to EdU/pH3/Aurora B-positive signal in HL-1 cell line. Data were represented as means ± SEM (n = 3 biological replicates; ***P* < 0.01, ****P* < 0.001). Scale bar: 50 µm and 20 µm. **h-j** HL-1 cell line was transfected with CCNL1 siRNA and corresponding control siRNA (CTL siRNA), and the CM proliferation was evaluated by immunofluorescence staining with EdU, pH3 and Aurora B. The arrows point to EdU/pH3/Aurora B-positive signal in HL-1 cell line. DAPI co-stains the nucleus. Data were represented as means ± SEM (n = 3 biological replicates; ****P* < 0.001). Scale bar: 50 and 20 µm.


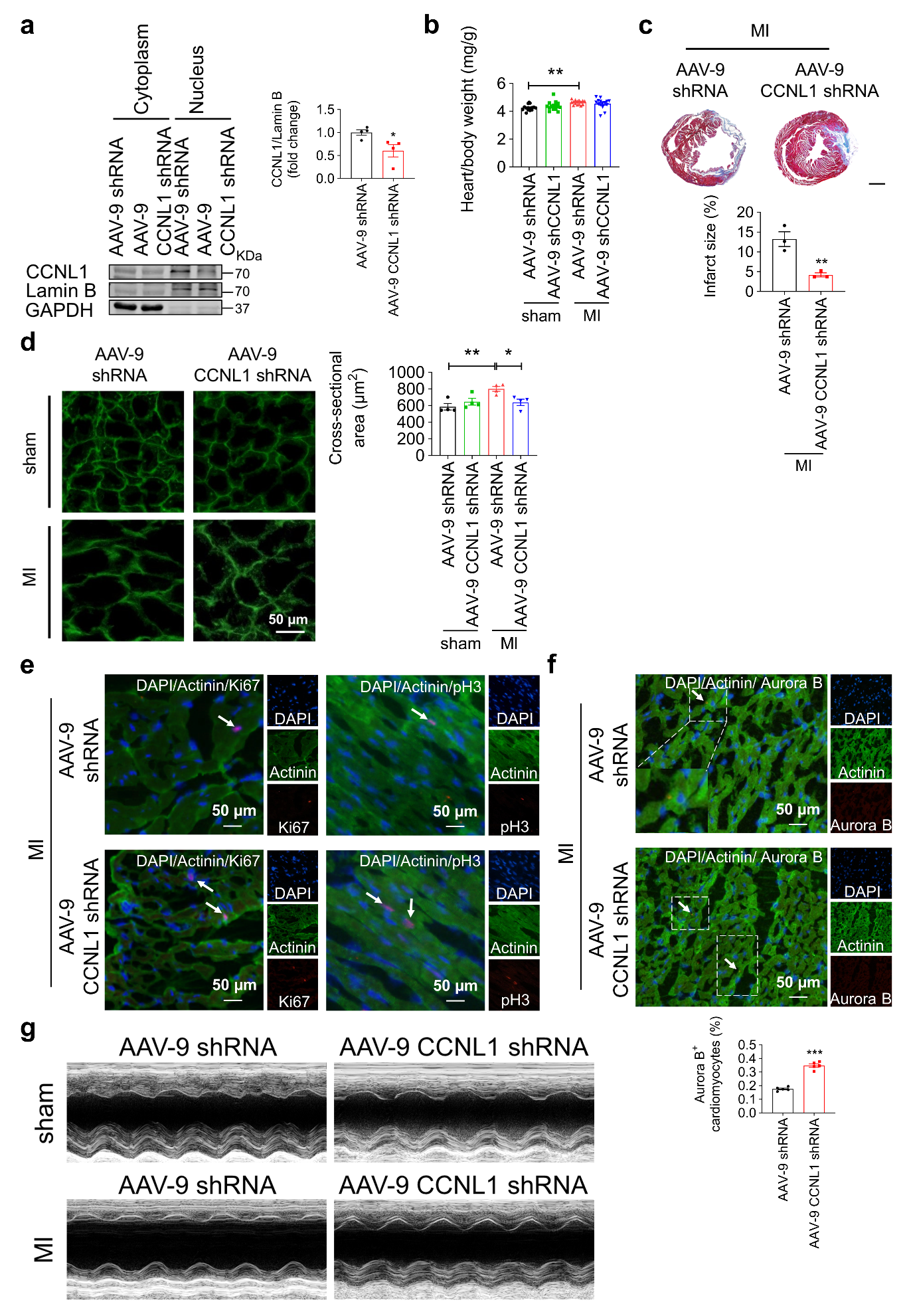


**Figure S3. The silencing of CCNL1 promotes heart repair after MI in adult mice. a** Western blot analysis of the silencing efficiency of AAV-9 CCNL1 shRNA in mice. Lamin B was served as a loading control for the nucleus and GAPDH for the cytoplasm. Data were represented as means ± SEM (n = 4; **P* < 0.05). **b** The heart weight to body weight ratio. ANOVA test was used (n = 15; ***P* < 0.01). **c** The infarct size of adult MI mice 28 days after administration of AAV-9 CCNL1 shRNA was evaluated by Masson trichrome staining. Data were represented as means ± SEM (n = 3; ***P* < 0.01). Scale bar: 500 µm. **d** The heart tissues of adult MI mice 28 days after administration of AAV-9 CCNL1 shRNA were stained with WGA. ANOVA test was used (n = 4; **P* < 0.05, ***P* < 0.01). **e** The representative image of Ki67/pH3 immunofluorescence staining in the heart tissue of MI mice 28 days after administration of AAV-9 CCNL1 shRNA. The arrows indicate Ki67/pH3-positive signal in CM. Scale bars: 50 µm. **f** The representative image of Aurora B immunofluorescence staining in the heart tissue of MI mice 28 days after administration of AAV-9 CCNL1 shRNA. The arrows indicate Aurora B-positive signal in CM. Data were represented as means ± SEM (n = 5; ****P* < 0.001). Scale bars: 50 µm. **g** The representative images of cardiac function in adult MI mice at 28 days after administration of AAV-9 CCNL1 shRNA were analyzed by echocardiography.

**
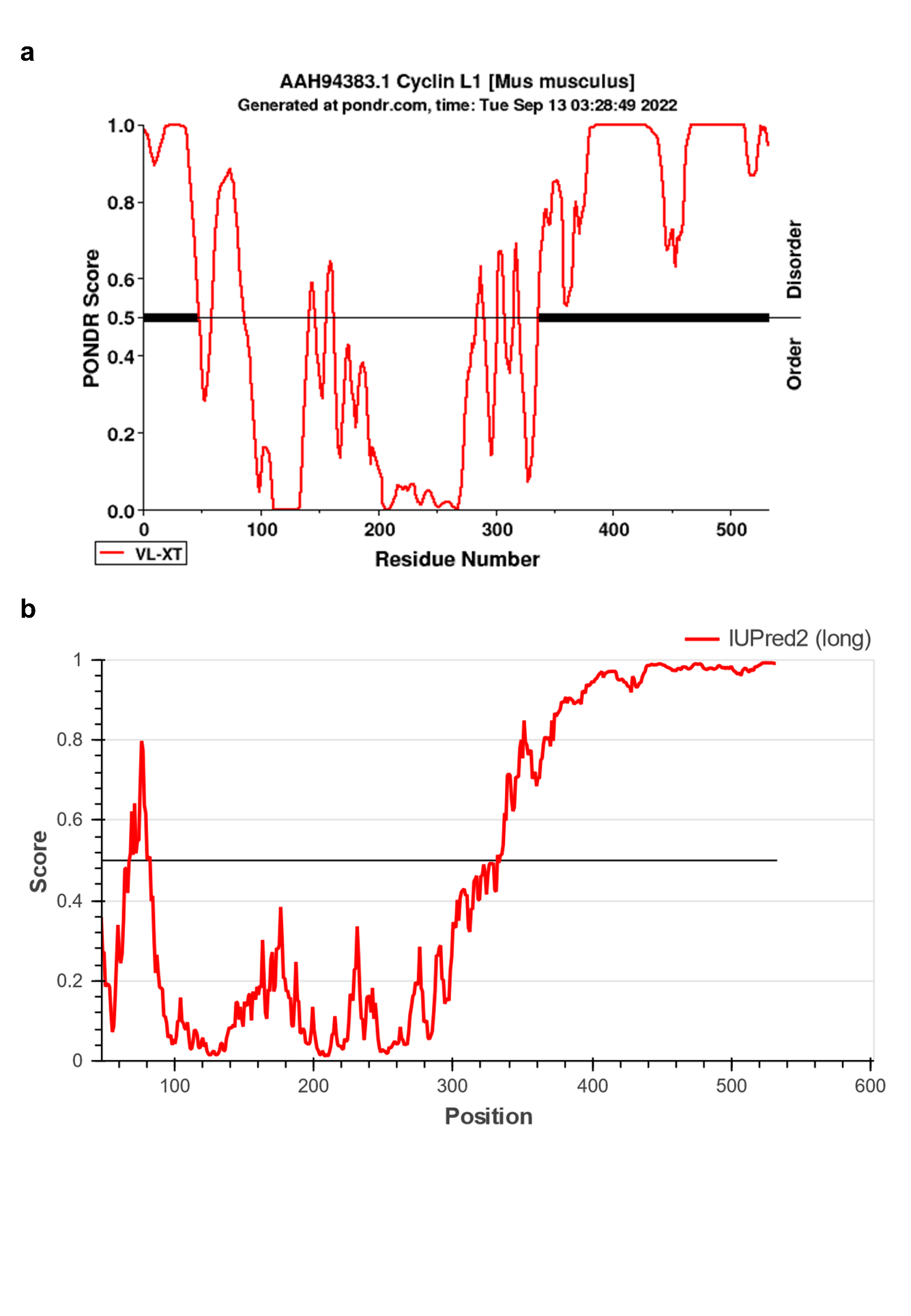
**

**Figure S4. a, b** Using the algorithm (PONDR (http://www.pondr.com/) and IUPred2A (https://iupred2a.elte.hu/plot_new) to predict the IDRs of full length CCNL1 protein.

**
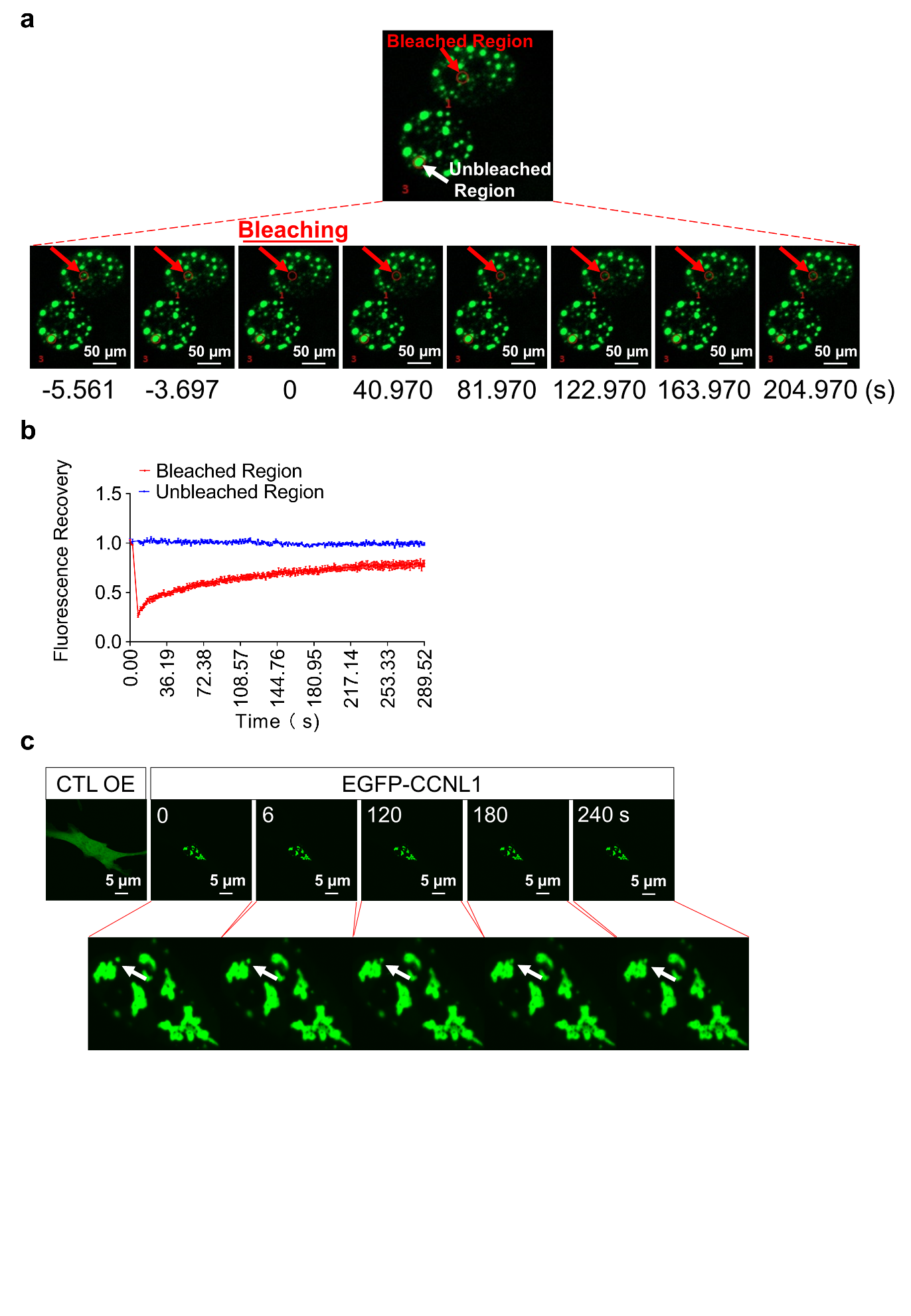
**

**Figure S5. The puncta of EGFP-CCNL1 in the nucleus of CM and HL-1** **cell line is highly dynamic and exhibits liquid properties.** **a** FRAP analysis of puncta of EGFP-CCNL1 formed over time in the nucleus of CM. 1: the region of photobleaching; 3: the region of un-photobleaching. Scale bars: 50 µm. **b** The HL-1 cell line was transfected with EGFP-CCNL1 for 48 h and FRAP was performed. Quantitative FRAP data are shown as mean ± SEM. A typical FRAP recovery curve of puncta of EGFP-CCNL1 averaged from n = 6 biological replicates. **c** Time-lapse imaging of the fusion behavior of puncta of EGFP-CCNL1 in the nucleus of HL-1 cell line. The time interval is 60 s. n = 3 biological replicates. Scale bars: 5 µm.

**
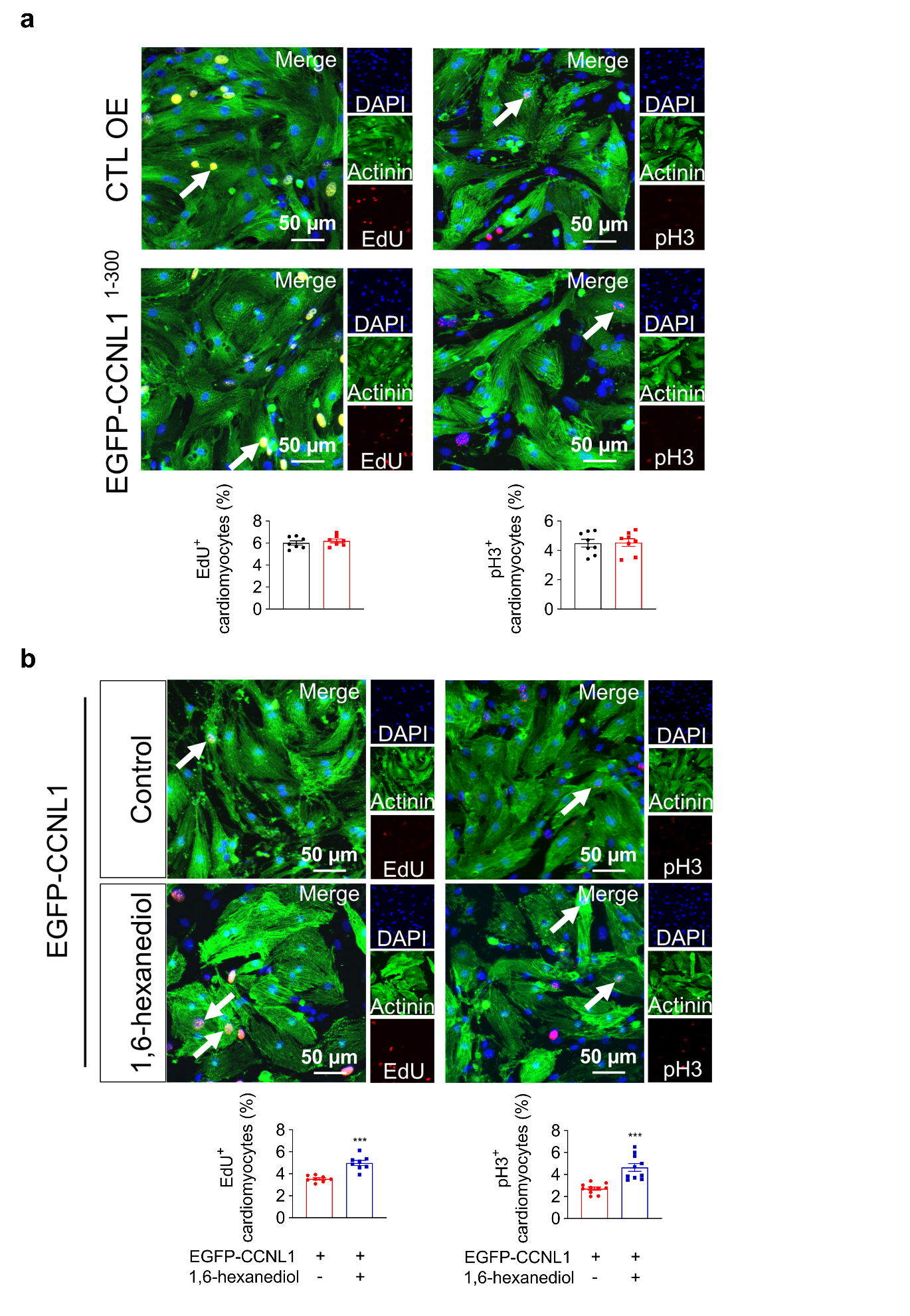
**

**Figure S6. The effect of LLPS behavior of CCNL1 on CM proliferation. a** The neonatal CM was transfected with CTL plasmid (CTL OE) and EGFP-CCNL1^1-300^ plasmid (EGFP-CCNL1^1-300^), and the CM proliferation was evaluated by immunofluorescence staining of EdU, pH3 and Actinin (marked CM). The arrows point to EdU/pH3-positive signal in CM. Data were represented as means ± SEM (EdU: n = 7; pH3: n = 8). Scale bar: 50 µm. **b** The neonatal CM was transfected with EGFP-CCNL1 and treated with 0.25% 1, 6-hexanediol, and the CM proliferation was evaluated by immunofluorescence staining of EdU, pH3 and Actinin (marked CM). The arrows point to EdU/pH3-positive signal in CM. DAPI co-stains the nucleus. Data were represented as means ± SEM (EdU: n = 8; pH3: n = 10; ****P* < 0.001). Scale bar: 50 µm.

**
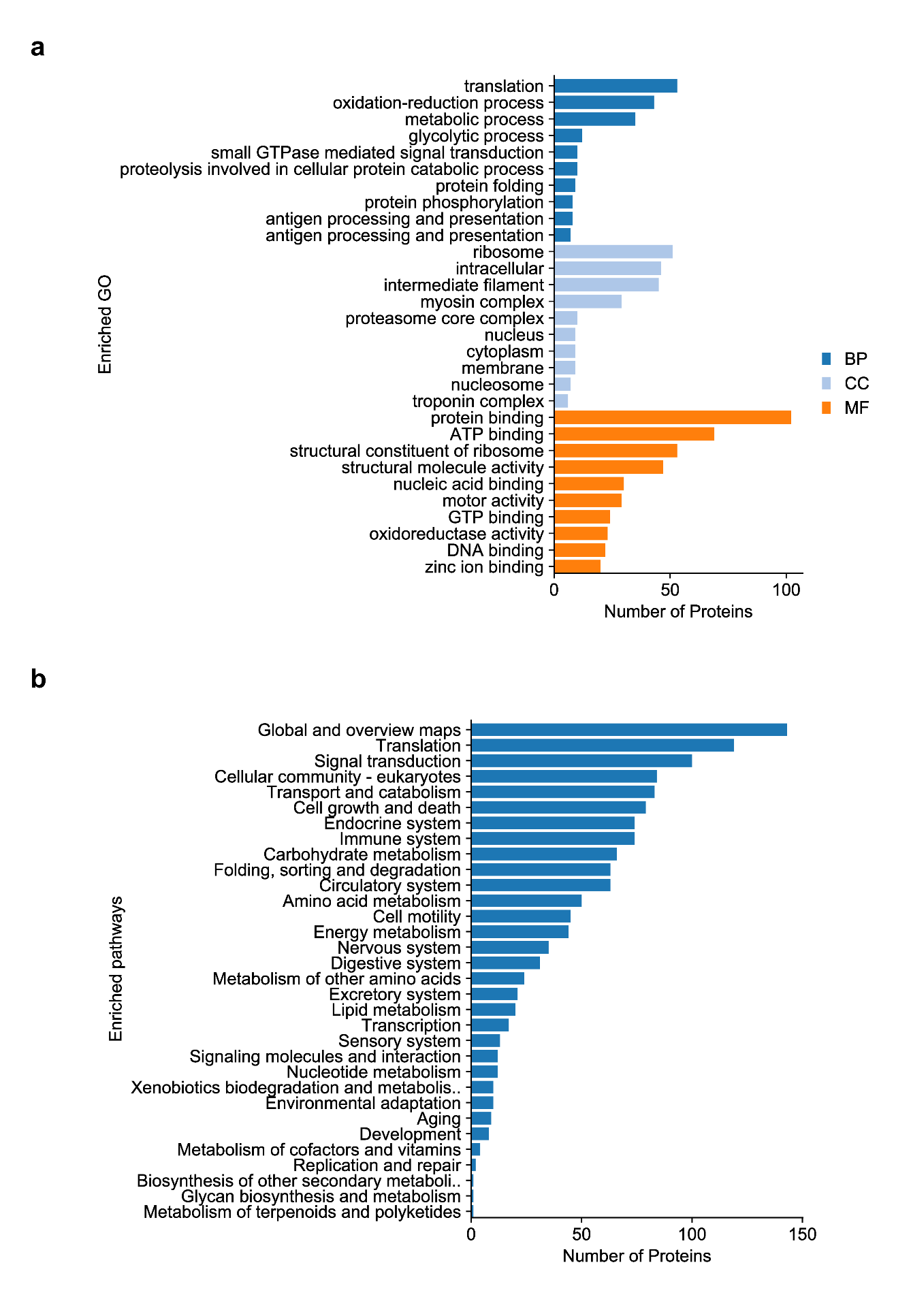
**

**Figure S7. Identification and classification of** **Co-IP/LC-MS analysis data. a, b** Gene Ontology (GO) and Kyoto Encyclopedia of Genes and Genomes (KEGG) pathway of proteins were analyzed using Co-IP/LC-MS analysis data.


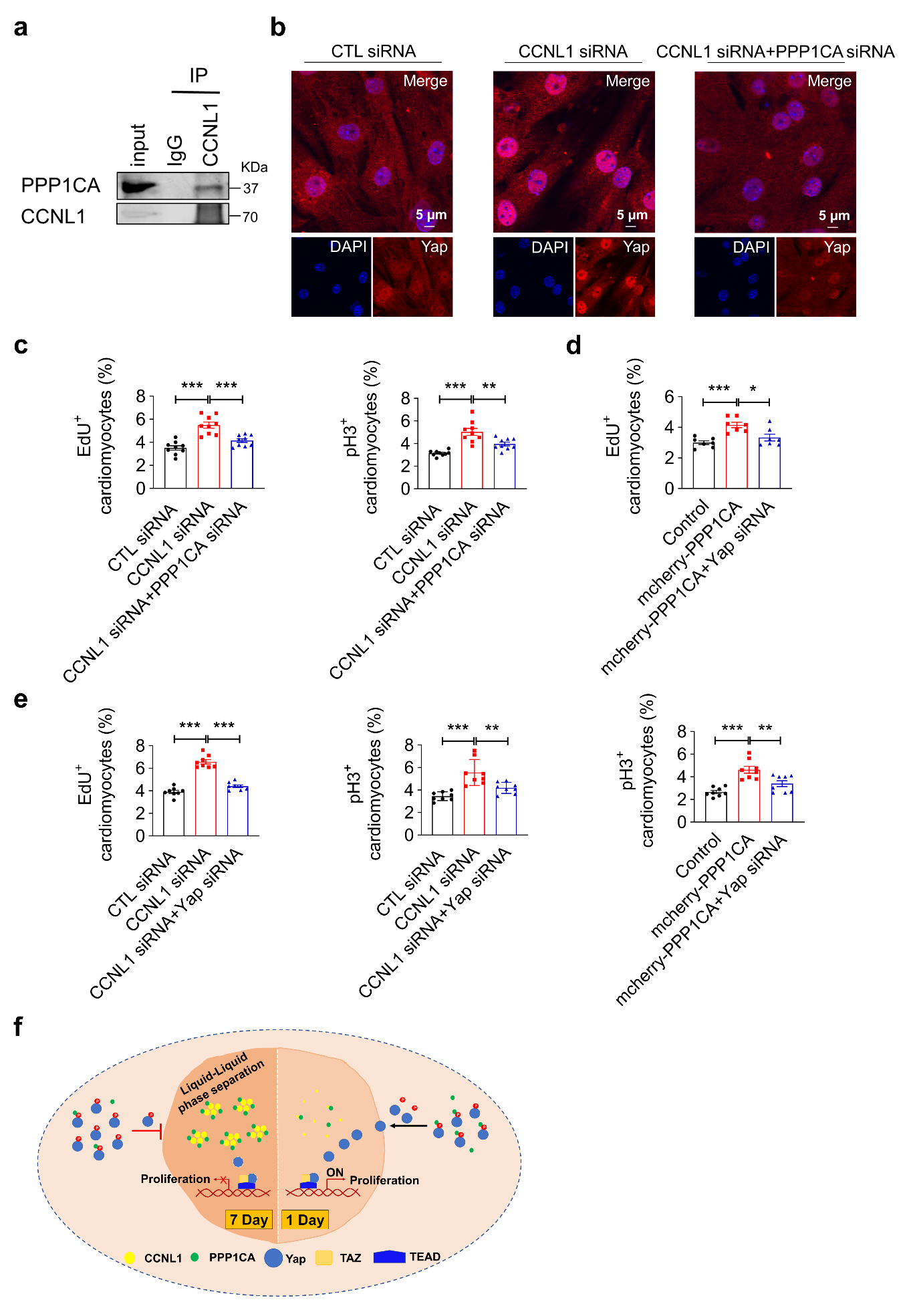


**Figure S8.** **CCNL1 inhibits CM proliferation, which is at least partially associated with CCNL1/PPP1CA nuclear accumulation and the less nuclear translocation of Yap. a** Co-immunoprecipitation (Co-IP) assay was performed in neonatal CM to investigate interaction between CCNL1 and PPP1CA. IgG was served as control. **b** The neonatal CM were co-transfected with CCNL1 siRNA and small interfering RNA-targeting PPP1CA (PPP1CA siRNA), and the localization of Yap in the nucleus and cytoplasm of CM was evaluated by immunofluorescence staining. n = 3 biological replicates. **c-e** The neonatal CM was transfected with small interfering RNA-targeting CCNL1, PPP1CA or Yap (CCNL1 siRNA, PPP1CA siRNA or Yap siRNA) and co-transfected with mcherry-PPP1CA plasmid and Yap siRNA, and the CM proliferation was evaluated by immunofluorescence staining of EdU and pH3. Data were represented as means ± SEM (n = 7-9; **P* < 0.05, ***P* < 0.01, ****P* < 0.001). **f** The elevated concentration of CCNL1 can undergo LLPS in the nucleus of CM after birth and inhibit CM proliferation.

**Supplementary Table**

**Table S1. SiRNA sequences.**

| siRNA | Sequence (5'-3') |
| --- | --- |
| Mus-CCNL1 siRNA  Mus-PPP1CA siRNA | 5'- AGAAUGUGAACGUAACCAATT-3'  5'- UUGGUUACGUUCACAUUCUTT-3'  5'-GAUGAAUGCAAGAGAAGAUTT-3'  5'-AUCUUCUClUUGCAUUCAUCTT-3' |
| Mus-Yap siRNA  CTL siRNA | 5'-GAUGAAUUCUGCCUCAGGATT-3'  5'-UCCUGAGGCAGAAUUCAUCTT-3'  5'- UUCUCCGAACGUGUCACGUTT-3'  5'- ACGUGACACGUUCGGAGAATT-3' |

CCNL1: Cyclin L1.

**Original result of Co-IP/LC-MS**

| Protein | Description | Gene | IgG_CCNL1 | IP_CCNL1 |
| --- | --- | --- | --- | --- |
| P35282 | Ras-related protein Rab-21 | Rab21 | 0 | - |
| Q5NC80 | Nucleoside diphosphate kinase (Fragment) | Nme1 | - | 615860.38 |
| P42208 | Septin-2 | Septin2 | 403111.688 | 0 |
| Q8BVU9 | Collagen-binding protein | Serpinh1 | 6464829.31 | 3100132.5 |
| P68369 | Tubulin alpha-1A chain | Tuba1a | 0 | 35203.465 |
| P14115 | 60S ribosomal protein L27a | Rpl27a | 1276134.25 | 2113558 |
| A0A1L1SQA8 | 40S ribosomal protein S25 | Rps25 | 22142312.5 | 16528843 |
| P84084 | ADP-ribosylation factor 5 | Arf5 | - | 508312.22 |
| P99026 | Proteasome subunit beta type-4 | Psmb4 | 4277533 | - |
| P99027 | 60S acidic ribosomal protein P2 | Rplp2 | 0 | 963323.44 |
| P99024 | Tubulin beta-5 chain | Tubb5 | 4871332.75 | 8494074.6 |
| Q9WUM5 | Succinate--CoA ligase [ADP/GDP-forming] subunit alpha, mitochondrial | Suclg1 | 15396380.1 | 19065196 |
| A0A2R8VHP3 | Predicted pseudogene 5478 | Gm5478 | 12843636.4 | 16211104 |
| P10126 | Elongation factor 1-alpha 1 | Eef1a1 | 0 | 11430105 |
| O70251 | Elongation factor 1-beta | Eef1b | 6856961.63 | - |
| A0A1L7NR37 | Arg/Abl-binding protein 2 | Sorbs2 | 4757406.28 | 17434298 |
| Q91V55 | 40S ribosomal protein S5 | Rps5 | 5700191.38 | 4857839.2 |
| Q91VT4 | 3-oxoacyl-[acyl-carrier-protein] reductase | Cbr4 | 0 | - |
| D6REV6 | F-box only protein 22 | Fbxo22 | 369419 | 455651.44 |
| Q3TYH2 | Ras-related protein Rab-15 | Rab15 | - | 2660085 |
| A0A2I3BR69 | Structural maintenance of chromosomes protein 1B (Fragment) | Smc1b | 1063249.63 | - |
| P68510 | 14-3-3 protein eta | Ywhah | 753050 | 918653.06 |
| Q8BSH3 | Tropomyosin alpha-1 chain | Tpm1 | - | 40615964 |
| P97379 | Ras GTPase-activating protein-binding protein 2 | G3bp2 | 0 | - |
| A0A068BGR9 | Complex I-B14.5a | Ndufa7 | - | 0 |
| Q9CZ13 | Cytochrome b-c1 complex subunit 1, mitochondrial | Uqcrc1 | 20031685.6 | 20926957 |
| Q9ES97 | Reticulon-3 | Rtn3 | 324109.563 | - |
| A0A2R8VK58 | Myoglobin (Fragment) | Mb | 1146368.25 | - |
| E9PUM3 | Alpha-1,4 glucan phosphorylase | Pygm | 654078.25 | 2399940.6 |
| Q9D051 | Pyruvate dehydrogenase E1 component subunit beta, mitochondrial | Pdhb | 23098419 | 9936228.5 |
| A0A4Y5WWJ7 | Myosin XVIIIA transcript variant 6 | Myo18a | - | 2953595.4 |
| A0A1S6GWH1 | AAA domain-containing protein | Psmc5 | 369619.969 | 6303834 |
| Q497D7 | 60S ribosomal protein L30 | Rpl30 | 3679216.06 | 0 |
| P17751 | Triosephosphate isomerase | Tpi1 | 71102948.8 | 54176194 |
| Q60597 | 2-oxoglutarate dehydrogenase, mitochondrial | Ogdh | 5882975.09 | 3409873.9 |
| Q5RKN9 | F-actin-capping protein subunit alpha | Capza1 | 0 | 3207297 |
| Q78ZJ8 | RAB11B protein | Rab11b | 684092.125 | - |
| A0A2R8VHY9 | Poly(rC)-binding protein 2 (Fragment) | Pcbp2 | 0 | - |
| P48962 | ADP/ATP translocase 1 | Slc25a4 | 28528682.6 | 26897750 |
| Q9JKB3 | Y-box-binding protein 3 | Ybx3 | 600026.688 | - |
| Q3TIJ9 | Uncharacterized protein | Actb | - | 5557482 |
| Q571F9 | MKIAA4115 protein (Fragment) | G3bp1 | - | 1638857.4 |
| Q3UAF6 | Uncharacterized protein | Actb | - | 0 |
| Q9DC70 | NADH dehydrogenase [ubiquinone] iron-sulfur protein 7, mitochondrial | Ndufs7 | 414349.219 | - |
| Q9DB77 | Cytochrome b-c1 complex subunit 2, mitochondrial | Uqcrc2 | 18850314.1 | 20102493 |
| Q9CZN7 | Serine hydroxymethyltransferase, mitochondrial | Shmt2 | 16009992.5 | 13721522 |
| P26041 | Moesin | Msn | 5941876.38 | 1536667.1 |
| Q1MWP8 | EH-domain containing 4-KJR (Fragment) | Ehd4 | 24863150.4 | 35386835 |
| Q3UBU0 | HATPase_c domain-containing protein | Hsp90b1 | 2478203.44 | 1619327.1 |
| Q543N3 | LIM and SH3 domain protein 1 | Lasp1 | 2558542.03 | - |
| F8WGM8 | Actin, cytoplasmic 2 (Fragment) | Actg1 | - | 784391 |
| Q5NCI4 | Phosphoglycerate mutase | Pgam2 | 1245155.75 | 1638635.5 |
| P62320 | Small nuclear ribonucleoprotein Sm D3 | Snrpd3 | 7377373.75 | 1662504.6 |
| Q3U4D1 | Adenosylhomocysteinase | | 0 | - |
| Q564G1 | Tropomyosin 1, alpha | Tpm1 | 56345325.7 | - |
| Q8BQM7 | Single-pass membrane and coiled-coil domain-containing protein 3 | Smco3 | 1310673.38 | - |
| Q497I3 | Fatty acid binding protein 5, epidermal | Fabp5 | 1105513.25 | 1145826.1 |
| A0A1B0GSS8 | 60S ribosomal protein L18 | Rpl18 | 1978563.75 | - |
| Q9DCT8 | Cysteine-rich protein 2 | Crip2 | 1224460.63 | 0 |
| D3YTZ8 | Ras-related protein Rab-15 | Rab15 | 2205647.75 | - |
| Q9DCT5 | Stromal cell-derived factor 2 | Sdf2 | 547399 | - |
| Q9D023 | Mitochondrial pyruvate carrier 2 | Mpc2 | 478512.719 | - |
| P51174 | Long-chain specific acyl-CoA dehydrogenase, mitochondrial | Acadl | 85948108 | 79583159 |
| Q9WVJ2 | 26S proteasome non-ATPase regulatory subunit 13 | Psmd13 | 436449.375 | 967763.97 |
| Q8CII2 | Cell division cycle protein 123 homolog | Cdc123 | 854867.625 | - |
| Q3UK30 | Uncharacterized protein | | 2268886.75 | - |
| Q5M9P3 | 40S ribosomal protein S19 (Fragment) | Rps19 | 111483763 | 45214713 |
| E9Q1G8 | Septin | Septin7 | 0 | 0 |
| Q8BKZ9 | Pyruvate dehydrogenase protein X component, mitochondrial | Pdhx | 2282448.88 | 2269684 |
| Q3TI61 | 26S proteasome non-ATPase regulatory subunit 2 | Psmd2 | 6990863.63 | 1813717.6 |
| Q3TMB8 | Adenylosuccinate lyase | Adsl | 1158534.38 | 592637.5 |
| Q61390 | T-complex protein 1 subunit zeta-2 | Cct6b | 0 | - |
| Q3UJS0 | 60S ribosomal protein L8 | Rpl8 | 2203058.16 | 1634872.6 |
| Q3U6S1 | Vimentin | Vim | 24572517.8 | 1160187.1 |
| Q9QWL7 | Keratin, type I cytoskeletal 17 | Krt17 | 6081188.5 | - |
| P62806 | Histone H4 | H4c1 | 12407030 | 9453701.9 |
| Q4FK88 | Annexin | Anxa1 | 498926.219 | - |
| Q3TE63 | Peptidyl-prolyl cis-trans isomerase | Ppia | 507892.281 | 848504.81 |
| Q3TF74 | Coronin | Coro1c | 4122476.06 | 4318869.7 |
| P62192 | 26S proteasome regulatory subunit 4 | Psmc1 | 517126.563 | - |
| A0A1W2P7T3 | Enhancer of rudimentary homolog | Erh | 0 | 0 |
| O70456 | 14-3-3 protein sigma | Sfn | 2314223.75 | - |
| Q5SW87 | RAB1A, member RAS oncogene family | Rab1a | - | 0 |
| Q9CQ69 | Cytochrome b-c1 complex subunit 8 | Uqcrq | 6595236.25 | 4236948 |
| Q80V84 | HtrA serine peptidase 2 | Htra2 | 0 | - |
| Q8BM63 | Uncharacterized protein (Fragment) | Prkag2 | 501906.156 | - |
| Q3THU8 | Phosphate carrier protein, mitochondrial | Slc25a3 | 10621691.7 | 7839475.5 |
| Q9D809 | RIKEN cDNA 2200002D01 gene | 2200002D01Rik | 114311.797 | - |
| Q3TKB9 | HATPase_c domain-containing protein (Fragment) | Hsp90aa1 | - | 891246.63 |
| Q9CQA3 | Succinate dehydrogenase [ubiquinone] iron-sulfur subunit, mitochondrial | Sdhb | 2280118.44 | 1500770.6 |
| Q3TFF0 | Uncharacterized protein | Dnaja2 | 3148591.06 | 1724337.4 |
| P16332 | Methylmalonyl-CoA mutase, mitochondrial | Mmut | 0 | 0 |
| Q5SX39 | Myosin-4 | Myh4 | 8195068.5 | 9260128 |
| A0A087WS46 | Eukaryotic translation elongation factor 1 beta 2 | Eef1b2 | - | 3540383.5 |
| D3YUM1 | NADH dehydrogenase [ubiquinone] flavoprotein 1, mitochondrial | Ndufv1 | 5265526.81 | 3146476.2 |
| Q2M4G1 | Metaxin 2 | Mtx2 | 0 | 0 |
| Q9D1M7 | Peptidyl-prolyl cis-trans isomerase FKBP11 | Fkbp11 | 3214215 | 0 |
| Q9D1M4 | Eukaryotic translation elongation factor 1 epsilon-1 | Eef1e1 | - | 0 |
| Q149V4 | Histone H2A | H2ac20 | 12835781.5 | - |
| Q1XH17 | Tripartite motif-containing protein 72 | Trim72 | 490793 | - |
| D3YYV8 | 60S ribosomal protein L5 (Fragment) | Rpl5 | 391150.406 | - |
| D3Z3Z9 | SH3 domain-binding glutamic acid-rich protein (Fragment) | Sh3bgr | 717724.625 | - |
| P26516 | 26S proteasome non-ATPase regulatory subunit 7 | Psmd7 | - | 589563.56 |
| Q9D8W5 | 26S proteasome non-ATPase regulatory subunit 12 | Psmd12 | 1441783.81 | - |
| P62715 | Serine/threonine-protein phosphatase 2A catalytic subunit beta isoform | Ppp2cb | 290563.5 | - |
| O08528 | Hexokinase-2 | Hk2 | 518430.375 | 630065.38 |
| O70325 | Phospholipid hydroperoxide glutathione peroxidase | Gpx4 | 4233557.44 | 923882 |
| Q99KV1 | DnaJ homolog subfamily B member 11 | Dnajb11 | 2120786.5 | 3056612.5 |
| Q3TIF8 | 40S ribosomal protein S24 | Rps24 | 7540674.5 | 0 |
| P80316 | T-complex protein 1 subunit epsilon | Cct5 | 4348215.25 | 5607000.9 |
| P80315 | T-complex protein 1 subunit delta | Cct4 | 12862908.3 | 10185245 |
| Q9D8E6 | 60S ribosomal protein L4 | Rpl4 | - | 639465.63 |
| A0A1L1SV25 | Alpha-actinin-4 | Actn4 | - | 883167.06 |
| P47911 | 60S ribosomal protein L6 | Rpl6 | 17502863.9 | 19761965 |
| Q05CM5 | Eif5 protein (Fragment) | Eif5 | 767062.938 | - |
| Q3TGJ9 | Actin-depolymerizing factor | Gsn | 0 | 1638634.1 |
| Q3U4H0 | APC-binding protein EB1 | Mapre1 | 549873.5 | 663158.31 |
| P06745 | Glucose-6-phosphate isomerase | Gpi | 18446951.5 | 2889765 |
| Q3UAZ3 | Arginine--tRNA ligase (Fragment) | Rars | 346281.563 | - |
| E9Q3Z5 | Supervillin | Svil | 3188925.84 | 3784179.9 |
| Q07417 | Short-chain specific acyl-CoA dehydrogenase, mitochondrial | Acads | 771237.375 | 766509.81 |
| E9QNY3 | Myosin regulatory light chain 10 | Myl10 | 885266.813 | 2340410 |
| Q61425 | Hydroxyacyl-coenzyme A dehydrogenase, mitochondrial | Hadh | 4916120.5 | 1188445.5 |
| Q9Z2I9 | Succinate--CoA ligase [ADP-forming] subunit beta, mitochondrial | Sucla2 | 6752293.8 | 6087323.5 |
| Q8C8G9 | Ecto-5-nucleotidase | | 569353.438 | - |
| P43274 | Histone H1.4 | H1-4 | 2424481.31 | - |
| Q07797 | Galectin-3-binding protein | Lgals3bp | 2461901.25 | 0 |
| Q9CXX7 | Uncharacterized protein | Snrpa | 2654044.75 | 966373.25 |
| Q3U9P7 | Succinyl-CoA:3-ketoacid-coenzyme A transferase | Oxct1 | 2717848.8 | 669297.25 |
| Q542X7 | CCT-beta | Cct2 | 6123332.69 | - |
| Q9CSU2 | 26S proteasome regulatory subunit RPN11 (Fragment) | Psmd14 | 0 | 0 |
| Q3TJD4 | ATP synthase subunit b | Atp5pb | 5823318.25 | 5212672 |
| Q9JI95 | CPN10-like protein | Hspe1-rs1 | 1773532.88 | 800463.75 |
| Q9JI91 | Alpha-actinin-2 | Actn2 | 9403658.81 | - |
| Q9D0R8 | Protein LSM12 homolog | Lsm12 | - | 0 |
| P29387 | Guanine nucleotide-binding protein subunit beta-4 | Gnb4 | 1494142 | 2172356.3 |
| A0A1W2P7A1 | 40S ribosomal protein S12 | Rps12 | 1675224.5 | 2436959.4 |
| Q3UHZ5 | Leiomodin-2 | Lmod2 | 56130614.5 | 73381501 |
| Q922B2 | Aspartate--tRNA ligase, cytoplasmic | Dars1 | 484181.875 | - |
| A0A571BDS0 | Xin actin-binding repeat-containing protein 1 | Xirp1 | 368675.531 | 487683.34 |
| Q923G3 | F-actin-capping protein subunit beta | Capzb | 2698553.63 | 4051571.6 |
| O54734 | Dolichyl-diphosphooligosaccharide--protein glycosyltransferase 48 kDa subunit | Ddost | 1151146.56 | - |
| Q9WTI7 | Unconventional myosin-Ic | Myo1c | 50675415.9 | 39507529 |
| Q3TSG8 | Uncharacterized protein | Trap1 | 5138868.5 | 4648462 |
| Q4V9X9 | Rpl23a protein (Fragment) | Rpl23a | 27431212.3 | 19311141 |
| Q9CZS1 | Aldehyde dehydrogenase X, mitochondrial | Aldh1b1 | 5552003.25 | 6096070.5 |
| E9PWE8 | Dihydropyrimidinase-related protein 3 | Dpysl3 | 1767012.13 | 1012655.5 |
| Q0VDM9 | Krt78 protein (Fragment) | Krt78 | 113595844 | 111038192 |
| Q6A0F1 | CCT-theta (Fragment) | Cct8 | 13898519.2 | 16578420 |
| F6QC77 | Troponin I, slow skeletal muscle (Fragment) | Tnni1 | 333121508 | 391165744 |
| Q3UEB3 | Poly(U)-binding-splicing factor PUF60 | Puf60 | 8152865.5 | 3886218.8 |
| B7FAV1 | Filamin, alpha (Fragment) | Flna | 1460993.13 | 6705022.5 |
| Q3TQP7 | Uncharacterized protein | Acat1 | 3454410.31 | - |
| Q6ZWV7 | 60S ribosomal protein L35 | Rpl35 | 13751580.3 | 9140396.6 |
| P62843 | 40S ribosomal protein S15 | Rps15 | 286671.656 | - |
| Q3TGW0 | Actin-like protein 3 | Actr3 | 4077735.13 | 1565292.4 |
| A0A1B0GS70 | Proteasome subunit alpha type-1 | Psma1 | 836652.25 | 2352270.4 |
| A2ARP8 | Microtubule-associated protein 1A | Map1a | 0 | - |
| Q3UIH5 | 26S proteasome AAA-ATPase subunit RPT1 | Psmc2 | 192742.453 | - |
| P61358 | 60S ribosomal protein L27 | Rpl27 | 10222800.8 | 8195760.3 |
| Q6ZWX6 | Eukaryotic translation initiation factor 2 subunit 1 | Eif2s1 | 5691969.66 | - |
| A0A0G2JEX1 | Nexilin | Nexn | - | 0 |
| O88783 | Coagulation factor V | F5 | 489663.938 | - |
| P05213 | Tubulin alpha-1B chain | Tuba1b | 0 | 0 |
| A0A1W2P7X3 | Acid phosphatase | Acp1 | - | 0 |
| P48787 | Troponin I, cardiac muscle | Tnni3 | 428447089 | 865531509 |
| Q3U7V7 | Profilin | Pfn1 | 1561891.81 | - |
| Q9CR57 | 60S ribosomal protein L14 | Rpl14 | 9649908.81 | 9457806.9 |
| Q9DCW4 | Electron transfer flavoprotein subunit beta | Etfb | 9983542.88 | 8860062.6 |
| A2AMI7 | Eukaryotic translation initiation factor 4 gamma 3 (Fragment) | Eif4g3 | 0 | - |
| P62317 | Small nuclear ribonucleoprotein Sm D2 | Snrpd2 | 4625234.56 | 3284773.4 |
| P62315 | Small nuclear ribonucleoprotein Sm D1 | Snrpd1 | 2549975 | 664294.5 |
| Q8C8R3 | Ankyrin-2 | Ank2 | 0 | - |
| Q3UV17 | Keratin, type II cytoskeletal 2 oral | Krt76 | 119485816 | 180596752 |
| P63268 | Actin, gamma-enteric smooth muscle | Actg2 | 72525801.9 | 682761328 |
| P21981 | Protein-glutamine gamma-glutamyltransferase 2 | Tgm2 | 3628432.05 | 4967719.6 |
| P59999 | Actin-related protein 2/3 complex subunit 4 | Arpc4 | 4574003.75 | 5234470.8 |
| P63260 | Actin, cytoplasmic 2 | Actg1 | 17854041.1 | 189031.78 |
| P61750 | ADP-ribosylation factor 4 | Arf4 | 1233627.75 | 1388129.9 |
| Q8JZQ9 | Eukaryotic translation initiation factor 3 subunit B | Eif3b | 306698.906 | - |
| Q920M5 | Coronin-6 | Coro6 | 4230977.5 | 18878552 |
| Q9CYL5 | Golgi-associated plant pathogenesis-related protein 1 | Glipr2 | 502382.969 | 1937797.4 |
| Q3TI99 | L-lactate dehydrogenase | Ldha | 95196958.8 | 94325280 |
| P17897 | Lysozyme C-1 | Lyz1 | 4836438.19 | 1456211.3 |
| Q9QUM9 | Proteasome subunit alpha type-6 | Psma6 | 1719599.88 | 0 |
| Q4FK74 | ATP synthase F1 subunit delta | Atp5d | 9101636.56 | 11942679 |
| E9Q405 | Unconventional myosin-XVIIIa | Myo18a | 2102915.81 | - |
| P09041 | Phosphoglycerate kinase 2 | Pgk2 | 1291657.5 | 780759.5 |
| Q99JY0 | Trifunctional enzyme subunit beta, mitochondrial | Hadhb | 24248447.8 | 31916607 |
| Q3UCL5 | Ferritin |  | 663095.688 | - |
| Q80U89 | MKIAA0034 protein (Fragment) | mKIAA0034 | 19242881.8 | 12969019 |
| P62141 | Serine/threonine-protein phosphatase PP1-beta catalytic subunit | Ppp1cb | 688133.875 | - |
| A0A0U1RNT6 | S-adenosylmethionine synthase | Mat2a | 0 | - |
| Q9QZ88 | Vacuolar protein sorting-associated protein 29 | Vps29 | 744894.75 | 747350.06 |
| O70468 | Myosin-binding protein C, cardiac-type | Mybpc3 | 1645206.41 | 420205.38 |
| E9Q9T1 | Leucine-rich repeat flightless-interacting protein 1 | Lrrfip1 | - | 1797448.3 |
| Q3TIU7 | NADH-ubiquinone oxidoreductase 75 kDa subunit, mitochondrial | Ndufs1 | 7366795.63 | 8434208.4 |
| P11031 | Activated RNA polymerase II transcriptional coactivator p15 | Sub1 | 1181083.25 | - |
| Q9R0X4 | Acyl-coenzyme A thioesterase 9, mitochondrial | Acot9 | 5477909.06 | 4021873.9 |
| Q6ZQ32 | MKIAA0858 protein (Fragment) | Lmo7 | 2348400.47 | - |
| P53026 | 60S ribosomal protein L10a | Rpl10a | 19704293 | 21467362 |
| O08583 | THO complex subunit 4 | Alyref | 4110541.38 | 1907283 |
| P10649 | Glutathione S-transferase Mu 1 | Gstm1 | 1721765.75 | - |
| Q505A8 | Ribosomal protein L39 | Rpl39 | 3723828.75 | - |
| Q9WV35 | C->U-editing enzyme APOBEC-2 | Apobec2 | 7694133.81 | 7326115.5 |
| Q3TNK2 | WD_REPEATS_REGION domain-containing protein | Wdr1 | 11442462.1 | 57824048 |
| P50543 | Protein S100-A11 | S100a11 | 1644040 | 1709026.5 |
| Q8VC94 | 60S ribosomal protein L11 | Rpl11 | 3123613.13 | 3995200.4 |
| B1ARW4 | Complex I-15 kDa (Fragment) | Ndufs5 | 1327698.13 | - |
| P50544 | Very long-chain specific acyl-CoA dehydrogenase, mitochondrial | Acadvl | 4512382 | 2716749.9 |
| Q3UHW9 | ADF-H domain-containing protein | Cfl2 | 7317555.25 | 10942683 |
| Q99L13 | 3-hydroxyisobutyrate dehydrogenase, mitochondrial | Hibadh | 495003.813 | 841834.19 |
| Q3U548 | UTP--glucose-1-phosphate uridylyltransferase | Ugp2 | 1126191.84 | - |
| P70670 | Nascent polypeptide-associated complex subunit alpha, muscle-specific form | Naca | 1666329.88 | 2890115.4 |
| Q9CQZ5 | NADH dehydrogenase [ubiquinone] 1 alpha subcomplex subunit 6 | Ndufa6 | 626885 | 0 |
| Q9WV55 | Vesicle-associated membrane protein-associated protein A | Vapa | 223655.953 | - |
| Q99LC5 | Electron transfer flavoprotein subunit alpha, mitochondrial | Etfa | 6098122 | 8214980.5 |
| Q5M8Q0 | Ribosomal protein L15 | Rpl15 | 894202.313 | 0 |
| Q99LC3 | NADH dehydrogenase [ubiquinone] 1 alpha subcomplex subunit 10, mitochondrial | Ndufa10 | 1296155.81 | 1677394.6 |
| Q9JKS4 | LIM domain-binding protein 3 | Ldb3 | 0 | - |
| Q9D7S7 | 60S ribosomal protein L22-like 1 | Rpl22l1 | 4436971.63 | 2708399.4 |
| Q3UCW4 | Cathepsin D | Ctsd | 1419860.63 | 1050055.3 |
| Q5YLW3 | 40S ribosomal protein S3 | Rps3 | 18103491.6 | 18735777 |
| P63038 | 60 kDa heat shock protein, mitochondrial | Hspd1 | 17904673.1 | 15335121 |
| A0A1S6GWI0 | Complex I-19kD | Ndufa8 | 1176546.31 | 1410236.4 |
| A8IP69 | 14-3-3 protein gamma subtype | Ywhag | 2134285.44 | 1775122.5 |
| Q3U4U6 | T-complex protein 1 subunit gamma | Cct3 | 14628846.6 | 9969217.5 |
| O35658 | Complement component 1 Q subcomponent-binding protein, mitochondrial | C1qbp | 592614.563 | 617848 |
| A2AEX6 | Four and a half LIM domains protein 1 | Fhl1 | - | 998704.5 |
| P49813 | Tropomodulin-1 | Tmod1 | 67144067.3 | 110320600 |
| E9Q1V0 | Hsc70-interacting protein (Fragment) | St13 | 0 | - |
| P97807 | Fumarate hydratase, mitochondrial | Fh | 13044282.8 | 25492205 |
| Q1WWK3 | Hist1h1b protein (Fragment) | H1f5 | - | 1010248.4 |
| P62960 | Y-box-binding protein 1 | Ybx1 | 5536753.75 | 8033646.9 |
| E9Q616 | AHNAK nucleoprotein (desmoyokin) | Ahnak | 1581223.94 | - |
| D3Z4S3 | Putative peptidyl-tRNA hydrolase PTRHD1 | Ptrhd1 | 255802.953 | - |
| A0A0G2JG59 | Nexilin (Fragment) | Nexn | - | 1796136.1 |
| Q3UJK5 | Ornithine--oxo-acid aminotransferase | Oat | - | 7692269.4 |
| Q3UYH9 | Alpha-1,4 glucan phosphorylase | Pygb | 985118.25 | - |
| Q543D7 | Uncharacterized protein | Fhl2 | 866843.938 | - |
| Q64475 | Histone H2B type 1-B | H2bc3 | 21690223.4 | 14696567 |
| Q9Z2U1 | Proteasome subunit alpha type-5 | Psma5 | 0 | 0 |
| Q641N3 | Rps16 protein (Fragment) | Rps16 | 5135062.25 | 5201834.1 |
| Q93092 | Transaldolase | Taldo1 | - | 398488 |
| Q61414 | Keratin, type I cytoskeletal 15 | Krt15 | 0 | - |
| P63101 | 14-3-3 protein zeta/delta | Ywhaz | 3371582 | 4038877.9 |
| Q542G9 | Annexin | Anxa2 | 6224930.47 | - |
| Q3TTM6 | Alpha-MPP | Pmpca | - | 0 |
| Q9CQP0 | 39S ribosomal protein L33, mitochondrial | Mrpl33 | 638656.438 | - |
| O09131 | Glutathione S-transferase omega-1 | Gsto1 | 3089963.66 | 1731211.9 |
| Q497N1 | 40S ribosomal protein S26 | Rps26 | 591013 | - |
| Q9ERS2 | NADH dehydrogenase [ubiquinone] 1 alpha subcomplex subunit 13 | Ndufa13 | 3359836.63 | 2891166.8 |
| Q8BTY3 | Inorganic diphosphatase | Ppa1 | 0 | - |
| P45952 | Medium-chain specific acyl-CoA dehydrogenase, mitochondrial | Acadm | 14775642.8 | 15973220 |
| P09542 | Myosin light chain 3 | Myl3 | 1901740594 | 3.566E+09 |
| A0A0G2JGN4 | Small nuclear ribonucleoprotein-associated protein B | Snrpb | - | 1450780.8 |
| P61164 | Alpha-centractin | Actr1a | 6368426.19 | 4060135.3 |
| Q3V2C6 | IF rod domain-containing protein | Des | 17113815.8 | 10520597 |
| Q45VK7 | Cytoplasmic dynein 2 heavy chain 1 | Dync2h1 | 510815.25 | - |
| P48774 | Glutathione S-transferase Mu 5 | Gstm5 | 639931.125 | - |
| Q3U1J4 | DNA damage-binding protein 1 | Ddb1 | 10148197 | - |
| Q3UJR8 | Transcription factor BTF3 | Btf3 | 1091060.31 | - |
| Q8BNA5 | SH3 domain-containing protein | Cttn | 0 | 0 |
| Q3TIQ2 | 60S ribosomal protein L12 | Rpl12 | - | 14412835 |
| Q9Z239 | Phospholemman | Fxyd1 | 0 | - |
| P46735 | Unconventional myosin-Ib | Myo1b | 482741.313 | - |
| P68134 | Actin, alpha skeletal muscle | Acta1 | 738867 | 1.219E+09 |
| Q91WD5 | NADH dehydrogenase [ubiquinone] iron-sulfur protein 2, mitochondrial | Ndufs2 | 3742940.63 | 1156896.6 |
| B1AWZ5 | Protein NipSnap homolog 3B | Nipsnap3b | 369868.375 | 1639599.1 |
| Q6RI64 | Proteasome subunit beta | Psmb1 | 1041123.03 | - |
| Q61495 | Desmoglein-1-alpha | Dsg1a | 267527.563 | 422795.56 |
| P34884 | Macrophage migration inhibitory factor | Mif | 17495813.3 | 18133550 |
| Q8BK51 | Alpha-MPP | Pmpca | 568154 | - |
| Q8BME2 | NADH dehydrogenase [ubiquinone] 1 alpha subcomplex subunit 12 | Ndufa12 | 1392018.03 | 1525901.5 |
| P16460 | Argininosuccinate synthase | Ass1 | 437143.813 | 0 |
| Q3UFJ3 | Pyruvate dehydrogenase E1 component subunit alpha | Pdha1 | 4596349.19 | 5858618.5 |
| P14152 | Malate dehydrogenase, cytoplasmic | Mdh1 | 7984155.88 | 7578058 |
| A0A087WS31 | Aspartyl aminopeptidase (Fragment) | Dnpep | 3435193.44 | 0 |
| P38647 | Stress-70 protein, mitochondrial | Hspa9 | 34935893.3 | 101474996 |
| A0A087WPK3 | Leucine-rich repeat flightless-interacting protein 1 | Lrrfip1 | 3904131.44 | - |
| P52480 | Pyruvate kinase PKM | Pkm | 95689696.4 | 92225961 |
| E9Q7U2 | Calcium-binding and coiled-coil domain-containing protein 1 | Calcoco1 | - | 1250582.3 |
| A0A140LJ59 | Eukaryotic translation initiation factor 3 subunit K | Eif3k | 4938036 | - |
| P62274 | 40S ribosomal protein S29 | Rps29 | 0 | - |
| A0A338P7A0 | NADH dehydrogenase [ubiquinone] iron-sulfur protein 4, mitochondrial (Fragment) | Ndufs4 | 1738398.75 | 1368089.3 |
| Q5FWB7 | Fructose-bisphosphate aldolase | Aldoa | 13460146.9 | 166026181 |
| Q5FWB6 | 60S acidic ribosomal protein P0 | Rplp0 | 27503902.3 | 27099532 |
| A0A338P6X5 | RNA helicase (Fragment) | Eif4a2 | - | 0 |
| P47753 | F-actin-capping protein subunit alpha-1 | Capza1 | 927441.188 | 1561755.5 |
| P47757 | F-actin-capping protein subunit beta | Capzb | 20998830 | 38009371 |
| Q9DCX2 | ATP synthase subunit d, mitochondrial | Atp5pd | 52273193.7 | 57753005 |
| P47758 | Signal recognition particle receptor subunit beta | Srprb | 331396.844 | - |
| E9Q919 | Dynactin subunit 3 | Dctn3 | 590772.625 | 804580.75 |
| Q3UZQ3 | Elongation factor 1-alpha | Eef1a1 | 842820.375 | - |
| Q9D0M3 | Cytochrome c1, heme protein, mitochondrial | Cyc1 | - | 7893296.5 |
| A0A0G2JDY6 | Nexilin (Fragment) | Nexn | 3964153.75 | 4093157.5 |
| Q9D0M5 | Dynein light chain 2, cytoplasmic | Dynll2 | 804696.688 | - |
| B0LAC7 | Myosin light polypeptide 6 alkali smooth muscle and non-muscle protein (Fragment) | Myl6 | 9522920 | 9447029 |
| Q3TN31 | Proteasome subunit alpha type | Psma7 | 632124.813 | 0 |
| Q5HZY7 | V-type proton ATPase subunit G | Atp6v1g1 | - | 0 |
| Q9JHJ0 | Tropomodulin-3 | Tmod3 | 24721819.4 | 52773295 |
| E9PV48 | Interferon-induced protein with tetratricopeptide repeats 3B | Ifit3b | 1421197.19 | 2837019.3 |
| P62631 | Elongation factor 1-alpha 2 | Eef1a2 | 7759301.75 | 7807118.3 |
| Q8VHX6 | Filamin-C | Flnc | 1352685.81 | 1055737 |
| E9PV44 | ATP synthase F1 subunit epsilon | Atpif1 | 0 | - |
| Q6GT24 | Peroxiredoxin-6 | Prdx6 | 0 | 0 |
| D6RGM2 | O-phosphoseryl-tRNA(Sec) selenium transferase | Sepsecs | 1654609.13 | 2009705 |
| G3X9T7 | Galectin-9 | Lgals9 | - | 599535.06 |
| Q3UY05 | Complex I-23kD | Ndufs8 | 0 | - |
| Q01853 | Transitional endoplasmic reticulum ATPase | Vcp | 1953221.5 | 0 |
| Q3U9Q3 | Heterogeneous nuclear ribonucleoprotein K | Hnrnpk | 0 | 506884.03 |
| Q8BH59 | Calcium-binding mitochondrial carrier protein Aralar1 | Slc25a12 | 0 | 0 |
| O54724 | Caveolae-associated protein 1 | Cavin1 | 550740.188 | - |
| Q3UBP6 | Uncharacterized protein | Actb | - | 0 |
| Q8K2B3 | Succinate dehydrogenase [ubiquinone] flavoprotein subunit, mitochondrial | Sdha | 25791296.4 | 23123963 |
| Q8VD62 | UPF0696 protein C11orf68 homolog | Bles03 | - | 268061.09 |
| Q9DBN7 | Uncharacterized protein | Eci1 | 2861477.94 | 2162520.5 |
| Q5FW97 | 2-phospho-D-glycerate hydro-lyase | EG433182 | 8174284.44 | 21237313 |
| Q9QZD9 | Eukaryotic translation initiation factor 3 subunit I | Eif3i | 5923548.06 | 4541713.3 |
| Q8CHW0 | Lancl1 protein (Fragment) | Lancl1 | 0 | - |
| A0A140T8T4 | 60S ribosomal protein L9 | Rpl9-ps6 | 3108342.63 | 3666789.2 |
| Q69ZX3 | MKIAA0866 protein (Fragment) | Myh11 | 4760655.75 | 593135.06 |
| Q9D172 | Glutamine amidotransferase-like class 1 domain-containing protein 3A, mitochondrial | Gatd3a | 0 | - |
| Q99KI0 | Aconitate hydratase, mitochondrial | Aco2 | 4689840.41 | 3385299.5 |
| A0A482CXK4 | MHC class I heavy chain heavy chain (Fragment) | H2-K | 0 | - |
| A0A4E9FT70 | IgG3 (Fragment) | IGHG3 | 414851.563 | - |
| E9Q1S3 | Protein transport protein SEC23 | Sec23a | 0 | - |
| A0A0G2JFD8 | Nexilin | Nexn | 28644168.2 | - |
| Q9CR68 | Cytochrome b-c1 complex subunit Rieske, mitochondrial | Uqcrfs1 | 3529760.72 | 2757004.5 |
| P02463 | Collagen alpha-1(IV) chain | Col4a1 | 0 | 0 |
| Q9DCM0 | Persulfide dioxygenase ETHE1, mitochondrial | Ethe1 | 390723.719 | - |
| Q52KG9 | Chaperonin containing Tcp1, subunit 6a (Zeta) | Cct6a | 6607934.56 | 7530556.4 |
| P05202 | Aspartate aminotransferase, mitochondrial | Got2 | 90438793.7 | 103301372 |
| P05201 | Aspartate aminotransferase, cytoplasmic | Got1 | 8322958.38 | 9299634.3 |
| Q32P04 | Keratin 5 | Krt5 | 78966103.1 | 58203419 |
| Q6ZWY3 | 40S ribosomal protein S27-like | Rps27l | 429479.594 | - |
| D3YUI7 | Myosin regulatory light chain 2, ventricular/cardiac muscle isoform (Fragment) | Myl2 | 674046.625 | 1761985.3 |
| B2RWW8 | Myosin, heavy polypeptide 8, skeletal muscle, perinatal | Myh8 | - | 0 |
| Q3UC32 | Actin-related protein 2/3 complex subunit 5 | Arpc5 | - | 1213648.8 |
| P70698 | CTP synthase 1 | Ctps1 | 3440104.38 | 2372832 |
| Q3TPZ5 | Uncharacterized protein | Dctn2 | 6758393.94 | 4094740.9 |
| P54071 | Isocitrate dehydrogenase [NADP], mitochondrial | Idh2 | 47408124.8 | 55812027 |
| Q9Z1D1 | Eukaryotic translation initiation factor 3 subunit G | Eif3g | 1854703.19 | 1426843.5 |
| P63094 | Guanine nucleotide-binding protein G(s) subunit alpha isoforms short | Gnas | 0 | - |
| Q3TWV0 | Vimentin | Vim | - | 192779.39 |
| A2BFF8 | Cytoplasmic dynein 1 intermediate chain 2 | Dync1i2 | 0 | 573292.88 |
| Q3U7N2 | MICOS complex subunit MIC60 | Immt | 10046368.6 | - |
| B1AU25 | Apoptosis-inducing factor 1, mitochondrial | Aifm1 | 323753.031 | - |
| Q9CZI7 | Annexin | Anxa2 | - | 5784359.3 |
| Q60692 | Proteasome subunit beta type-6 | Psmb6 | 0 | 2770503.1 |
| P43276 | Histone H1.5 | H1-5 | 467318.75 | - |
| Q9ESP1 | Stromal cell-derived factor 2-like protein 1 | Sdf2l1 | 311665.781 | 500622.28 |
| A0A0N4SUQ1 | Plasminogen activator inhibitor 1 RNA-binding protein | Serbp1 | 0 | 0 |
| Q99PT1 | Rho GDP-dissociation inhibitor 1 | Arhgdia | 2355836.75 | 18548852 |
| P00493 | Hypoxanthine-guanine phosphoribosyltransferase | Hprt1 | 7101540 | 6795063.9 |
| Q9Z1R9 | Protease, serine 1 (trypsin 1) | Prss1 | 6496316.5 | - |
| P09671 | Superoxide dismutase [Mn], mitochondrial | Sod2 | 8427944.63 | 34710995 |
| Q99JX4 | Eukaryotic translation initiation factor 3 subunit M | Eif3m | - | 752827.38 |
| Q6ZQ33 | MKIAA0857 protein (Fragment) | Rab11fip5 | 0 | - |
| Q9DCD0 | 6-phosphogluconate dehydrogenase, decarboxylating | Pgd | 436774.688 | 718179.06 |
| P05132 | cAMP-dependent protein kinase catalytic subunit alpha | Prkaca | 3515519.31 | 3801698.9 |
| P00405 | Cytochrome c oxidase subunit 2 | Mtco2 | 788067.438 | 939734 |
| A2AAW9 | Eukaryotic translation initiation factor 2 subunit 3, X-linked | Eif2s3x | - | 526321.81 |
| P68040 | Receptor of activated protein C kinase 1 | Rack1 | 14378983.7 | 14438939 |
| Q3UJU5 | 40S ribosomal protein S3a | Rps3a1 | 16380606.6 | 9970092.9 |
| Q8C2S9 | Major vault protein | Mvp | 14234906.9 | - |
| F8WIV2 | Serine (or cysteine) peptidase inhibitor, clade B, member 6a | Serpinb6a | 1207119.63 | - |
| P63325 | 40S ribosomal protein S10 | Rps10 | 10115433.6 | 5340228.3 |
| E9Q5F6 | Polyubiquitin-C (Fragment) | Ubc | 18076995.5 | 22499811 |
| A0A494BBA8 | 40S ribosomal protein S30 | Fau | 3461834 | 1804987.5 |
| Q8VHD0 | Breast cancer 2 | Brca2 | 11901792 | - |
| Q62426 | Cystatin-B | Cstb | 488135.344 | 0 |
| Q62425 | Cytochrome c oxidase subunit NDUFA4 | Ndufa4 | 7937176 | 3595356.3 |
| Q5M9N6 | Rpl37a protein | Rpl37a | 4034620.5 | 3972044.9 |
| Q91VA7 | Isocitrate dehydrogenase [NAD] subunit, mitochondrial | Idh3b | 8059764 | 6639103.1 |
| Q6IRT4 | Eukaryotic translation initiation factor 3 subunit F | Eif3f | 5442960.38 | 4256265.4 |
| E9PZF0 | Nucleoside diphosphate kinase | Gm20390 | 43204921.3 | 7692099.8 |
| Q9CQ19 | Myosin regulatory light polypeptide 9 | Myl9 | 11006429.1 | 29092961 |
| E9Q264 | Myosin, heavy chain 15 | Myh15 | 1069316 | 838952.63 |
| Q9CXW3 | Calcyclin-binding protein | Cacybp | 0 | - |
| Q02257 | Junction plakoglobin | Jup | 2372742.25 | 5439952.9 |
| A1BN54 | Alpha actinin 1a | Actn1 | 5435700.94 | 3941154.9 |
| Q6PAC1 | Actin-depolymerizing factor | Gsn | 2637183.25 | 6571356.5 |
| Q9CPQ8 | ATP synthase subunit g, mitochondrial | Atp5mg | 2119235.13 | - |
| Q9CPQ9 | Fructose-bisphosphate aldolase | Aldoart1 | 7818292.5 | - |
| B2RXT5 | Glucose-6-phosphate isomerase | Gpi1 | - | 1276589.5 |
| Q8CD23 | Nucleolin | Ncl | 6505225.69 | 4900225.4 |
| Q9CPQ1 | Cytochrome c oxidase subunit 6C | Cox6c | 1953356.5 | - |
| Q8BH95 | Enoyl-CoA hydratase, mitochondrial | Echs1 | 9894836.91 | 13792994 |
| Q9JJI8 | 60S ribosomal protein L38 | Rpl38 | 2468457.75 | 1414032 |
| F8WJ41 | 40S ribosomal protein S15a (Fragment) | Rps15a | 4075906.56 | 4854910 |
| F8WID5 | Tropomyosin alpha-1 chain | Tpm1 | - | 600273.5 |
| O55142 | 60S ribosomal protein L35a | Rpl35a | 1336586.75 | 2356016.3 |
| O55143 | Sarcoplasmic/endoplasmic reticulum calcium ATPase 2 | Atp2a2 | 12830067.7 | 10103411 |
| P19157 | Glutathione S-transferase P 1 | Gstp1 | 25330294.3 | 22879293 |
| Q3TET0 | T-complex protein 1 subunit eta | Cct7 | 11491894.9 | 9796210.9 |
| B7ZP22 | Heterogeneous nuclear ribonucleoproteins A2/B1 | Hnrnpa2b1 | 820550.313 | - |
| P97315 | Cysteine and glycine-rich protein 1 | Csrp1 | - | 190915.22 |
| Q3TXN0 | PDZ domain-containing protein (Fragment) | Htra2 | - | 842062.44 |
| Q4FZH2 | Ribosomal protein L26 | Rpl26 | 21061771.8 | 19586706 |
| P56480 | ATP synthase subunit beta, mitochondrial | Atp5f1b | 225481222 | 134853770 |
| A0A571BEC9 | Perilipin-4 | Plin4 | 3687083.06 | 1833631.3 |
| Q9Z2K1 | Keratin, type I cytoskeletal 16 | Krt16 | 2815497.31 | 2637198.3 |
| Q5RKP3 | 60S ribosomal protein L13 | Rpl13 | 8981122.5 | 9507097.3 |
| Q6URW6 | Myosin-14 | Myh14 | 35165974.3 | 35791655 |
| Q6S390 | Plectin 4 | Plec | 0 | 22810818 |
| P29268 | CCN family member 2 | Ccn2 | 1754360.56 | 2098531.3 |
| Q810V5 | Sfpq protein (Fragment) | Sfpq | - | 724022.06 |
| Q9D8N0 | Elongation factor 1-gamma | Eef1g | 20120775.6 | 18879668 |
| Q60737 | Casein kinase II subunit alpha | Csnk2a1 | 3465826.56 | 0 |
| A2AFQ2 | 3-hydroxyacyl-CoA dehydrogenase type-2 | Hsd17b10 | 3909017.63 | 5012325.1 |
| P19123 | Troponin C, slow skeletal and cardiac muscles | Tnnc1 | 70862892.6 | 123196437 |
| O70194 | Eukaryotic translation initiation factor 3 subunit D | Eif3d | 0 | - |
| E9Q1W0 | Calcium/calmodulin-dependent protein kinase | Camk2d | 4270439.66 | 2405816.1 |
| P26443 | Glutamate dehydrogenase 1, mitochondrial | Glud1 | 8388525.81 | 9550643.5 |
| Q3U381 | Zinc finger protein 692 | Znf692 | 0 | - |
| Q3U7K7 | E3 ubiquitin-protein ligase TRIM21 | Trim21 | 36410036.9 | 10870269 |
| Q3U1B7 | SERPIN domain-containing protein | Serpina3h | 0 | 0 |
| Q8BMS1 | Trifunctional enzyme subunit alpha, mitochondrial | Hadha | 38351907.7 | 22313572 |
| P60335 | Poly(rC)-binding protein 1 | Pcbp1 | 558377.188 | 1403148.9 |
| Q9JHI5 | Isovaleryl-CoA dehydrogenase, mitochondrial | Ivd | 630780.438 | 2178906.4 |
| Q7TQ48 | Sarcalumenin | Srl | 1237113.88 | 0 |
| A0A140LIZ5 | 26S proteasome AAA-ATPase subunit RPT3 | Psmc4 | 437578.375 | 0 |
| Q58ET1 | 60S ribosomal protein L7a | Rpl7a | 3630908.63 | 4994736.3 |
| P05125 | Natriuretic peptides A | Nppa | 0 | - |
| E9Q175 | Unconventional myosin-6 | Myo6 | 2280463.94 | 1481991.4 |
| Q9CZU6 | Citrate synthase, mitochondrial | Cs | 35679592.4 | 45112179 |
| Q61782 | Type I epidermal keratin mRNA, 3end (Fragment) | | 2800882.75 | 2552569.5 |
| P11983 | T-complex protein 1 subunit alpha | Tcp1 | 7401343.06 | 6420618.9 |
| Q91VB8 | Alpha globin 1 | Hba-a1 | 22361481.6 | - |
| Q5DQJ3 | F-actin-capping protein subunit alpha | Capza2 | 22469343.4 | 43970931 |
| Q9D2G2 | Dihydrolipoyllysine-residue succinyltransferase component of 2-oxoglutarate dehydrogenase complex, mitochondrial | Dlst | 14209590.4 | 10752694 |
| E9Q557 | Desmoplakin | Dsp | 6753146.98 | 19861544 |
| K3W4R7 | Troponin T, cardiac muscle | Tnnt2 | 677729042 | 1.359E+09 |
| Q8QZT1 | Acetyl-CoA acetyltransferase, mitochondrial | Acat1 | - | 7257958.6 |
| Q4VA28 | 60S ribosomal protein L21 | Rpl21 | 4944470.88 | 1546981.5 |
| Q4VA29 | RNA transcription, translation and transport factor protein | Rtraf | 1258539.84 | - |
| Q3UR88 | NTF2 domain-containing protein | G3bp1 | 1525426.63 | - |
| Q3UZI0 | Uncharacterized protein (Fragment) | Hnrnpr | 511700.094 | 0 |
| Q8VDD5 | Myosin-9 | Myh9 | 677586430 | 582112749 |
| Q3TZ89 | Protein transport protein Sec31B | Sec31b | 0 | 0 |
| A2A6U5 | Septin-9 (Fragment) | Septin9 | - | 0 |
| B2RUC7 | Serine/threonine kinase receptor associated protein | Strap | 1808928 | - |
| Q3UIJ2 | Protein-synthesizing GTPase | Eif2s3x | 8019430.47 | - |
| Q3UIJ3 | Uncharacterized protein | Actc1 | 1860492 | - |
| F7CJS8 | 40S ribosomal protein S9 (Fragment) | Rps9 | 3033900.25 | 4181933.7 |
| Q99LF8 | Polyadenylate-binding protein | Pabpc4 | 3243063.5 | 3017676 |
| Q3U962 | Collagen alpha-2(V) chain | Col5a2 | - | 0 |
| E9Q2Q9 | SH3 domain-binding glutamic acid-rich protein (Fragment) | Sh3bgr | - | 0 |
| F6WHQ7 | Glutathione S-transferase Mu 1 (Fragment) | Gstm1 | - | 4192506.5 |
| P14148 | 60S ribosomal protein L7 | Rpl7 | 1210761.81 | 4101862.4 |
| Q9CRS5 | Uncharacterized protein (Fragment) | Ewsr1 | 0 | - |
| P19783 | Cytochrome c oxidase subunit 4 isoform 1, mitochondrial | Cox4i1 | 2279457.88 | 0 |
| Q8CAQ8 | MICOS complex subunit Mic60 | Immt | - | 8672140.6 |
| Q4VAG4 | Ribosomal protein L22 | Rpl22 | 21864938 | 13914638 |
| Q3UB67 | Uncharacterized protein | Rpl3 | - | 1487062.8 |
| Q9CVB6 | Actin-related protein 2/3 complex subunit 2 | Arpc2 | 4700725.19 | 2310862.8 |
| Q3TGZ3 | Isocitrate dehydrogenase [NAD] subunit, mitochondrial | Idh3g | 2804412.19 | 1658768.5 |
| A0A286YE28 | Transketolase (Fragment) | Tkt | 448145.469 | - |
| Q3UC02 | 40S ribosomal protein S11 | Rps11 | 8711034.88 | 22555587 |
| Q5SW83 | Uncharacterized protein | Actr2 | 5061121.81 | 9287545.4 |
| P08730 | Keratin, type I cytoskeletal 13 | Krt13 | - | 218195930 |
| A0A0A6YX05 | Sodium/potassium-transporting ATPase subunit beta (Fragment) | Atp1b1 | - | 2780208.5 |
| A0A1B0GSF0 | 60S ribosomal protein L13a | Rpl13a | 694371.25 | 998520.81 |
| Q99LP6 | GrpE protein homolog 1, mitochondrial | Grpel1 | 0 | - |
| P10639 | Thioredoxin | Txn | 0 | 0 |
| P47915 | 60S ribosomal protein L29 | Rpl29 | - | 5013803.8 |
| Q8BMF4 | Dihydrolipoyllysine-residue acetyltransferase component of pyruvate dehydrogenase complex, mitochondrial | Dlat | 21984556.7 | 21970549 |
| H7BX95 | Serine/arginine-rich splicing factor 1 | Srsf1 | 2760551.25 | - |
| P29341 | Polyadenylate-binding protein 1 | Pabpc1 | 3853135.88 | 3728941.5 |
| Q3TTY5 | Keratin, type II cytoskeletal 2 epidermal | Krt2 | 99307339.6 | 142133049 |
| E9Q9C7 | Actin-binding LIM protein 1 | Ablim1 | 517378.469 | - |
| Q5SX53 | Uncharacterized protein | Slc25a11 | 642346.625 | - |
| P11404 | Fatty acid-binding protein, heart | Fabp3 | 5108391.13 | 4616163.8 |
| Q62261 | Spectrin beta chain, non-erythrocytic 1 | Sptbn1 | 9989680.28 | 16978703 |
| Q6P5E4 | UDP-glucose:glycoprotein glucosyltransferase 1 | Uggt1 | 357727.188 | - |
| O35459 | Delta(3,5)-Delta(2,4)-dienoyl-CoA isomerase, mitochondrial | Ech1 | 1202051.66 | - |
| A0A1L1STE6 | Isocitrate dehydrogenase [NAD] subunit, mitochondrial | Idh3a | 1284790.31 | 1332621.5 |
| A0A6I8MX27 | L-lactate dehydrogenase | Ldhb | 28926616.1 | 33773776 |
| P27048 | Small nuclear ribonucleoprotein-associated protein B | Snrpb | 3016543.5 | - |
| A2A702 | Eukaryotic translation initiation factor 3 subunit M | Eif3m | 946477.063 | - |
| Q99L45 | Eukaryotic translation initiation factor 2 subunit 2 | Eif2s2 | 3471243.81 | - |
| Q9D898 | Actin-related protein 2/3 complex subunit 5-like protein | Arpc5l | 392264.344 | - |
| P62242 | 40S ribosomal protein S8 | Rps8 | 1520563.25 | 490447.03 |
| Q545C7 | Uncharacterized protein | Csrp3 | 0 | 2465093.1 |
| A6H644 | Protein phosphatase 1 regulatory subunit | Ppp1r12b | - | 0 |
| D3YZ80 | Interferon-induced protein with tetratricopeptide repeats 2 (Fragment) | Ifit2 | 445933.875 | - |
| Q8BWT1 | 3-ketoacyl-CoA thiolase, mitochondrial | Acaa2 | 25280637.9 | 19232761 |
| A0A1W2P6F6 | Myosin light polypeptide 6 | Myl6 | 121453085 | 150868230 |
| Q80T06 | Elongation factor 1-delta | Eef1d | 18129538.4 | 21350823 |
| Q3TK56 | Actin-related protein 2/3 complex subunit 5 (Fragment) | Arpc5 | 887850.875 | - |
| P67778 | Prohibitin | Phb | 7329911.63 | 4895048.3 |
| P68372 | Tubulin beta-4B chain | Tubb4b | 11054302 | - |
| A0A7N9VR94 | AHNAK nucleoprotein 2 | Ahnak2 | 0 | 0 |
| Q8VCQ8 | Caldesmon 1 | Cald1 | 5847968.03 | 17355894 |
| P61205 | ADP-ribosylation factor 3 | Arf3 | 0 | - |
| E9PZ67 | Calsequestrin (Fragment) | Casq2 | 1667719.88 | - |
| Q3UIB5 | Uncharacterized protein | Dhrs4 | - | 0 |
| A8DUP0 | Beta-globin | Hbbt1 | 14029392.6 | 5529568.9 |
| B0LAB0 | Eukaryotic translation elongation factor 1 epsilon 1 (Fragment) | Eef1e1 | 1104174.63 | - |
| P27546 | Microtubule-associated protein 4 | Map4 | 462833.875 | - |
| Q8CD98 | PFK domain-containing protein | Pfkl | 0 | - |
| P14069 | Protein S100-A6 | S100a6 | 1873964.13 | - |
| F8WHP8 | ATP synthase membrane subunit f | Atp5j2 | 0 | 0 |
| Q52L78 | Alpha(B)-crystallin | Cryab | 151379007 | 141726074 |
| Q91YN9 | BAG family molecular chaperone regulator 2 | Bag2 | 2178572 | 3462656.1 |
| Q91YR1 | Twinfilin-1 | Twf1 | 0 | 1942112.9 |
| Q8BFZ3 | Beta-actin-like protein 2 | Actbl2 | 19052302 | 34739173 |
| Q4FZE6 | 40S ribosomal protein S7 | Rps7 | 13441352.5 | 16209883 |
| Q3TRJ1 | Vacuolar protein sorting-associated protein 35 | Vps35 | - | 627888.19 |
| P51660 | Peroxisomal multifunctional enzyme type 2 | Hsd17b4 | 1040608.59 | 830191.38 |
| Q6ZWQ9 | Myosin, light chain 12A, regulatory, non-sarcomeric | Myl12a | 82558864.8 | 160723340 |
| F8VPN2 | Testis-expressed protein 15 | Tex15 | 2156380.25 | - |
| P51667 | Myosin regulatory light chain 2, ventricular/cardiac muscle isoform | Myl2 | 94068662.8 | 196367143 |
| Q5BLK2 | 40S ribosomal protein S20 | Rps20 | 17776884.5 | 13899462 |
| Q5BLK0 | 60S ribosomal protein L12 | Rpl12 | 16180968.9 | - |
| F7BAC9 | Catenin beta-1 (Fragment) | Ctnnb1 | 480684.719 | - |
| P51881 | ADP/ATP translocase 2 | Slc25a5 | 23044011.6 | 11358030 |
| O35129 | Prohibitin-2 | Phb2 | 4766349.06 | 7706754.5 |
| Q9EQK5 | Major vault protein | Mvp | - | 15698739 |
| Q5M9N5 | 60S ribosomal protein L28 | Rpl28 | 12748873.8 | 10632984 |
| D3YTP8 | U6 snRNA-associated Sm-like protein LSm4 (Fragment) | Lsm4 | 1314032.38 | - |
| Q6PCW9 | Myl1 protein | Myl1 | 0 | - |
| Q5M9N9 | Prdx2 protein | Prdx2 | 2039309.88 | 2262119.2 |
| Q3TL53 | 40S ribosomal protein S6 | | 619279.688 | 1524002.1 |
| P07901 | Heat shock protein HSP 90-alpha | Hsp90aa1 | 1963677.19 | - |
| P47738 | Aldehyde dehydrogenase, mitochondrial | Aldh2 | 1184338.56 | 1973659.5 |
| O09161 | Calsequestrin-2 | Casq2 | - | 2710073.5 |
| G3UZJ4 | Thioredoxin-dependent peroxiredoxin | Prdx5 | 3807059.75 | 5250523.6 |
| Q3UMI7 | Tr-type G domain-containing protein | Eef2 | 1665467.44 | 1416270.3 |
| Q8BFR5 | Elongation factor Tu, mitochondrial | Tufm | 3974366.88 | 1557132 |
| P16546 | Spectrin alpha chain, non-erythrocytic 1 | Sptan1 | - | 55033985 |
| P84244 | Histone H3.3 | H3-3a | 1470335.25 | 1307263.9 |
| Q571M2 | Heat shock 70 kDa protein 4 (Fragment) | Hspa4 | 2087355.13 | - |
| Q8BGH2 | Sorting and assembly machinery component 50 homolog | Samm50 | 1025401.88 | 328758.28 |
| Q99LF4 | RNA-splicing ligase RtcB homolog | Rtcb | 596971.063 | - |
| Q9DBR7 | Protein phosphatase 1 regulatory subunit 12A | Ppp1r12a | 558343.938 | 1370316.8 |
| Q8C290 | Uncharacterized protein | Hnrnpu | - | 3266316 |
| Q8VED5 | Keratin, type II cytoskeletal 79 | Krt79 | - | 0 |
| Q3TXS9 | 40S ribosomal protein S2 | Rps2 | 1171569.88 | 5120493 |
| E9Q0B5 | Fc fragment of IgG-binding protein | Fcgbp | 71444496 | 104366048 |
| P10833 | Ras-related protein R-Ras | Rras | 534386.813 | - |
| P62900 | 60S ribosomal protein L31 | Rpl31 | 12648402 | 17121661 |
| Q9JJD8 | CCT-beta | Cct2 | - | 4826057.3 |
| Q6PJ18 | Tpm2 protein | Tpm2 | 6275171.69 | - |
| Q3UAA9 | Uncharacterized protein | Actb | 0 | 0 |
| Q921R2 | 40S ribosomal protein S13 | Rps13 | 15824100.1 | 14340364 |
| Q6ZWZ4 | 60S ribosomal protein L36 | Rpl36 | 864135.125 | 1013168.6 |
| Q3TZK4 | MICOS complex subunit MIC60 (Fragment) | Immt | - | 52487242 |
| P15864 | Histone H1.2 | H1-2 | - | 6345555.3 |
| A2A513 | Keratin, type I cytoskeletal 10 | Krt10 | 166173173 | 273978998 |
| Q4FJR9 | G1p2 protein | Isg15 | 7852028.5 | 11032121 |
| P62869 | Elongin-B | Elob | 1097959.67 | - |
| Q8QZY1 | Eukaryotic translation initiation factor 3 subunit L | Eif3l | 7816690.63 | 4375637 |
| Q9D9P1 | MICOS complex subunit Mic19 | Chchd3 | 1649826.63 | 8699623.4 |
| Q5EBI8 | ATP synthase subunit e, mitochondrial | Atp5k | 2351122.25 | 0 |
| A0PJF4 | Ahnak protein (Fragment) | Ahnak | - | 1332223.1 |
| P14685 | 26S proteasome non-ATPase regulatory subunit 3 | Psmd3 | 0 | - |
| Q78IK2 | ATP synthase membrane subunit K, mitochondrial | Atp5mk | 572364.313 | - |
| Q9CQR4 | Acyl-coenzyme A thioesterase 13 | Acot13 | 3083579.13 | 1981710.6 |
| P24369 | Peptidyl-prolyl cis-trans isomerase B | Ppib | 17555504.8 | 0 |
| P62830 | 60S ribosomal protein L23 | Rpl23 | 3420009.13 | 1189351.6 |
| A0A1D5RME4 | 60S ribosomal protein L18a (Fragment) | Rpl18a | 725440.125 | 982747.31 |
| A0A3Q4L347 | Cysteine dioxygenase | Cdo1 | 1423146.75 | - |
| Q9CY06 | GLOBIN domain-containing protein | | - | 11383532 |
| A0A1L1SSC0 | Leucine-rich repeat flightless-interacting protein 2 | Lrrfip2 | 3501764 | 9196291.2 |
| Q3TEL1 | Uncharacterized protein | Psma3 | 0 | 0 |
| Q9CT23 | PCI domain-containing protein (Fragment) | Eif3e | - | 2311384.8 |
| Q64282 | Interferon-induced protein with tetratricopeptide repeats 1 | Ifit1 | 2815355.84 | 3214530.8 |
| Q545F8 | 40S ribosomal protein S4 | Rps4x | 24358772.3 | 13333569 |
| Q7M754 | Try10-like trypsinogen | Gm5409 | 18074298.9 | 15824976 |
| Q545F4 | Heat shock 27 kDa protein | Hspb1 | 26023598.2 | 26214815 |
| Q9JJ28 | Protein flightless-1 homolog | Flii | 4928601.84 | 30493144 |
| Q3UH59 | Myosin-10 | Myh10 | 187052379 | 298846269 |
| P08249 | Malate dehydrogenase, mitochondrial | Mdh2 | 51014313.8 | 50178443 |
| Q9CPY7 | Cytosol aminopeptidase | Lap3 | 6444800.06 | 4426957 |
| Q4FK59 | 2-phospho-D-glycerate hydro-lyase | Eno3 | 15671915.3 | 16662832 |
| B2RQQ1 | Myosin, heavy polypeptide 6, cardiac muscle, alpha | Myh6 | 6523512976 | 8.299E+09 |
| Q3UIG0 | Eukaryotic translation initiation factor 3 subunit E | Eif3e | 7053488.31 | - |
| P18760 | Cofilin-1 | Cfl1 | 8094059.56 | 7648667.9 |
| Q9R0Y5 | Adenylate kinase isoenzyme 1 | Ak1 | 1162928.75 | - |
| Q3TD78 | NIPSNAP domain-containing protein | Nipsnap2 | 11594795.2 | 9659515.8 |
| S4R1W7 | Ras-related protein Rab-35 | Rab35 | 2942675.75 | - |
| A0A0R3P9C8 | NADH dehydrogenase [ubiquinone] 1 alpha subcomplex subunit 9, mitochondrial | Ndufa9 | 2749206.47 | - |
| Q3TML0 | Protein disulfide-isomerase A6 | Pdia6 | 1460325.75 | 1335530.9 |
| Q8CF71 | Uncharacterized protein | Acta2 | 4115368.13 | 1602363.9 |
| O70569 | Ribosomal protein S14 | rps14 | 4012764.75 | 5755958.3 |
| Q3U8W0 | Serine/threonine-protein phosphatase PP1-alpha catalytic subunit | Ppp1ca | 0 | 430143.75 |
| O08749 | Dihydrolipoyl dehydrogenase, mitochondrial | Dld | 4232097.5 | 4319075.9 |
| Q9R0P5 | Destrin | Dstn | 2855111.5 | 2644206.9 |
| Q9R0P3 | S-formylglutathione hydrolase | Esd | 5608783 | 1773083.3 |
| Q60932 | Voltage-dependent anion-selective channel protein 1 | Vdac1 | 863325.5 | - |
| O89112 | Glutathione S-transferase LANCL1 | Lancl1 | - | 981574.44 |
| Q9CZ19 | Myosin light chain 4 | Myl4 | 50767075.6 | 159719717 |
| Q9DCL9 | Multifunctional protein ADE2 | Paics | 645358.5 | 695524.75 |
| Q3TXF9 | Sodium/potassium-transporting ATPase subunit alpha | Atp1a1 | 6017641.25 | 4253510.2 |
| Q8BMK4 | Cytoskeleton-associated protein 4 | Ckap4 | 30148314.8 | 42385985 |
| P62897 | Cytochrome c, somatic | Cycs | 2669794.25 | 1294152.1 |
| Q546G4 | Serum albumin | Alb | 11536341 | 7073840 |
| Q3TEK2 | Uncharacterized protein | Hspa8 | 78254369.9 | 88492558 |
| Q9CQ62 | 2,4-dienoyl-CoA reductase [(3E)-enoyl-CoA-producing], mitochondrial | Decr1 | 831289.453 | 917903 |
| Q5I0T8 | Ribosomal protein L19 | Rpl19 | 872326.25 | 2371534.3 |
| F2Z471 | Voltage-dependent anion-selective channel protein 1 | Vdac1 | - | 0 |
| B9EKJ1 | Spna2 protein | Sptan1 | 383939587 | - |
| Q9CQ65 | S-methyl-5-thioadenosine phosphorylase | Mtap | 2108608.81 | 777843.88 |
| Q3ULS8 | DNA damage-binding protein 1 (Fragment) | Ddb1 | - | 0 |
| A3KGE7 | Collagen alpha-1(V) chain (Fragment) | Col5a1 | 664001.438 | 2215622.4 |
| E9Q456 | Tropomyosin alpha-1 chain | Tpm1 | 31627518 | 0 |
| Q5SQB7 | Nucleophosmin | Npm1 | 566798.938 | - |
| A0A0A0MQM0 | Eukaryotic translation initiation factor 5A (Fragment) | Eif5a | 595277.438 | - |
| Q9D1R9 | 60S ribosomal protein L34 | Rpl34 | - | 4804373 |
| P20029 | Endoplasmic reticulum chaperone BiP | Hspa5 | - | 657263230 |
| Q6NXH9 | Keratin, type II cytoskeletal 73 | Krt73 | 0 | 0 |
| Q58E35 | Ribosomal protein, large, P1 | Rplp1 | 521591.906 | - |
| Q3U7Z6 | Phosphoglycerate mutase | Pgam1 | 1889900.69 | 0 |
| P53395 | Lipoamide acyltransferase component of branched-chain alpha-keto acid dehydrogenase complex, mitochondrial | Dbt | - | 793418.31 |
| Q7TMM9 | Tubulin beta-2A chain | Tubb2a | 2776203.25 | 5074862.5 |
| Q61781 | Keratin, type I cytoskeletal 14 | Krt14 | - | 6026579.9 |
| Q9CPP6 | NADH dehydrogenase [ubiquinone] 1 alpha subcomplex subunit 5 | Ndufa5 | 1921086.56 | - |
| Q6PFA2 | Clathrin light chain | Clta | 0 | - |
| Q3TJ21 | Pyrroline-5-carboxylate reductase | Pycr2 | 569656.188 | - |
| Q3TL95 | Proteasome subunit alpha type (Fragment) | Psma4 | - | 767233.44 |
| P04104 | Keratin, type II cytoskeletal 1 | Krt1 | 97713776 | 112195072 |
| A0A0G2JGY8 | 60S ribosomal protein L34 (Fragment) | Rpl34 | 2946189.91 | - |
| Q3THW7 | Eukaryotic translation initiation factor 3 subunit H | Eif3h | 2001594.69 | 1880709.5 |
| Q3THW5 | Histone H2A.V | H2az2 | - | 4194200.5 |
| Q549A5 | Clusterin | Clu | 3412222.88 | 2941592.5 |
| Q3UXC2 | RNA helicase | Eif4a1 | 2029715 | - |
| Q05CG9 | 26S proteasome non-ATPase regulatory subunit 1 (Fragment) | Psmd1 | 725828.125 | - |
| Q8BT90 | 40S ribosomal protein S17 (Fragment) | Rps17 | 16091713 | 10642137 |
| A0A494BAP3 | Ferritin (Fragment) | Fth1 | 2228460.06 | 2281681 |
| A0A0R4J023 | Methylglutaconyl-CoA hydratase, mitochondrial | Auh | 0 | - |
| Q3U292 | H15 domain-containing protein | H1f3 | 1597017.38 | - |
| A0A097PUD0 | Anti-dectin-1 15E2 light chain | | 0 | - |
| Q1A602 | Non-muscle alpha-actinin 4 | Actn4 | 1580951.19 | - |
| Q9CUS2 | Uncharacterized protein (Fragment) | Rsu1 | 1300698.25 | 1256435 |
| P14206 | 40S ribosomal protein SA | Rpsa | 2976431.38 | 6383094.3 |
| P57759 | Endoplasmic reticulum resident protein 29 | Erp29 | 0 | - |
| E9Q634 | Unconventional myosin-Ie | Myo1e | - | 0 |
| Q5FW75 | Actinin alpha 2 | Actn2 | - | 11162737 |
| O55125 | Protein NipSnap homolog 1 | Nipsnap1 | 0 | - |
| Q03265 | ATP synthase subunit alpha, mitochondrial | Atp5f1a | 218260763 | 183438312 |
| Q8C8T6 | Uncharacterized protein (Fragment) | | 844674.375 | - |
| Q3TJG6 | CS domain-containing protein | Ptges3 | 0 | - |
| A0A2R8W6S4 | Protein kinase C and casein kinase substrate in neurons protein 2 (Fragment) | Pacsin2 | 0 | - |
| Q9QYB1 | Chloride intracellular channel protein 4 | Clic4 | 1303216 | 438335.78 |
| Q3UNQ0 | Adenylyl cyclase-associated protein | Cap1 | 664228.875 | 0 |
| A2AIM4 | Tropomyosin beta chain | Tpm2 | - | 9935493 |
| Q9CQV8 | 14-3-3 protein beta/alpha | Ywhab | - | 0 |
| F6T4M4 | Serine/arginine repetitive matrix protein 1 (Fragment) | Srrm1 | 365095392 | - |
| D3Z4Z0 | Transcriptional adapter | Tada2b | - | 1110273.1 |
| Q61941 | NAD(P) transhydrogenase, mitochondrial | Nnt | - | 0 |
| Q61753 | D-3-phosphoglycerate dehydrogenase | Phgdh | 589755 | 0 |
| Q71LX8 | Heat shock protein 84b | Hsp90ab1 | 13183904.9 | 8561525.9 |
| Q04447 | Creatine kinase B-type | Ckb | 7382489.06 | 20472710 |
| Q9JK42 | [Pyruvate dehydrogenase (acetyl-transferring)] kinase isozyme 2, mitochondrial | Pdk2 | 313032.281 | - |
| Q3V2E0 | Peptidase S1 domain-containing protein | Try5 | 1374428.38 | - |
| A0A0A0MQF6 | Glyceraldehyde-3-phosphate dehydrogenase | Gapdh | 274209743 | 366525711 |
| A0A0R4J1P2 | Tropomyosin alpha-3 chain | Tpm3 | - | 580959.19 |
| Q3UW40 | TRASH domain-containing protein | Rpl24 | 3902990.63 | 2740631.8 |
| B2RXX9 | Myosin, heavy polypeptide 7, cardiac muscle, beta | Myh7 | 691708383 | 818965903 |
| C0HKD9 | Microfibrillar-associated protein 1B | Mfap1b | 352307.219 | - |
| Q3TV47 | Sodium/potassium-transporting ATPase subunit beta | Atp1b1 | 3873101.56 | - |
| P35700 | Peroxiredoxin-1 | Prdx1 | 4522158.69 | 5922795.9 |
| P70349 | Histidine triad nucleotide-binding protein 1 | Hint1 | 782712.75 | - |
| G5E8R1 | Tropomyosin alpha-1 chain | Tpm1 | 0 | 0 |
| P09103 | Protein disulfide-isomerase | P4hb | 746667.875 | 2057816.8 |
| Q8C1T2 | Uncharacterized protein | Psmd6 | 0 | 8573260 |
| Q4FJQ2 | Farsla protein | Farsa | 0 | - |
| Q3UKT3 | Ornithine--oxo-acid aminotransferase | Oat | 5973994.63 | - |
| A0A0N4SVK8 | [Protein ADP-ribosylarginine] hydrolase-like protein 1 | Adprhl1 | 6277305.97 | 7960097.1 |
| P07310 | Creatine kinase M-type | Ckm | 13657266.4 | 10417199 |
| Q9DB20 | ATP synthase subunit O, mitochondrial | Atp5po | 77797861.3 | 59816039 |
| Q9CWV1 | DNA helicase MCM8 | Mcm8 | 711835.25 | - |
| A0A2R8W6Z9 | Collagen, type XXII, alpha 1 | Col22a1 | - | 3816774.5 |
| Q561N5 | 40S ribosomal protein S18 | Rps18 | 75597724.1 | 86264733 |
| P52785 | Retinal guanylyl cyclase 1 | Gucy2e | 3206693.5 | - |
| Q8K4K8 | Antioxidant protein | | 4529470.78 | 4339663.9 |
| E0CYV0 | Protein-L-isoaspartate O-methyltransferase | Pcmt1 | - | 545838.5 |
| Q9CV24 | Uncharacterized protein (Fragment) | Eif2s1 | - | 1069692.8 |
| A0A2R8VHK1 | Cytochrome c1, heme protein, mitochondrial (Fragment) | Cyc1 | 6253494.25 | - |
| O89079 | Coatomer subunit epsilon | Cope | 0 | 979924.19 |
| Q9DCT2 | NADH dehydrogenase [ubiquinone] iron-sulfur protein 3, mitochondrial | Ndufs3 | 8309196.13 | 9867292.8 |
| Q54AH9 | Beta-2-globin (Fragment) | Hbb-b2 | - | 997897.81 |
| Q6IFX2 | Keratin, type I cytoskeletal 42 | Krt42 | 16152232.1 | 9951811.8 |
| Q5SX40 | Myosin-1 | Myh1 | 0 | - |
| Q8VIJ6 | Splicing factor, proline- and glutamine-rich | Sfpq | 367649.938 | - |
| Q3ULW0 | GTP-binding nuclear protein Ran | Ran | 1184153.03 | - |
| Q5BKQ9 | Proteasome (Prosome, macropain) 26S subunit, non-ATPase, 11 | Psmd11 | 1521818.31 | 1113014.6 |
| Q3TWF2 | 78 kDa glucose-regulated protein | Hspa5 | 482260098 | - |
| O08553 | Dihydropyrimidinase-related protein 2 | Dpysl2 | 1669740.63 | 861854.06 |
| P27773 | Protein disulfide-isomerase A3 | Pdia3 | 365214.094 | 0 |
| A0A1L1SUN1 | 60S ribosomal protein L29 (Fragment) | Rpl29 | 2126274.25 | - |
| Q8JZN5 | Complex I assembly factor ACAD9, mitochondrial | Acad9 | 0 | - |
| G3UX26 | Outer mitochondrial membrane protein porin 2 | Vdac2 | 3596978.59 | - |
| Q3TF84 | Uncharacterized protein | Lrrc59 | 8201848.81 | 2470380.5 |
| P62259 | 14-3-3 protein epsilon | Ywhae | 5114148.13 | 3822239.2 |
| Q6IME9 | Keratin, type II cytoskeletal 72 | Krt72 | 1239622.13 | 4204970 |
| F6TFN2 | LIM domain only 7 (Fragment) | Lmo7 | - | 5610381.9 |
| Q8C2Q8 | ATP synthase subunit gamma | Atp5c1 | 29829429.5 | 25178378 |
| P47857 | ATP-dependent 6-phosphofructokinase, muscle type | Pfkm | 413448.219 | - |
| P50446 | Keratin, type II cytoskeletal 6A | Krt6a | 3811594.75 | 1060129.3 |

**Original films of Western Blot**

**Figure 1:**

**
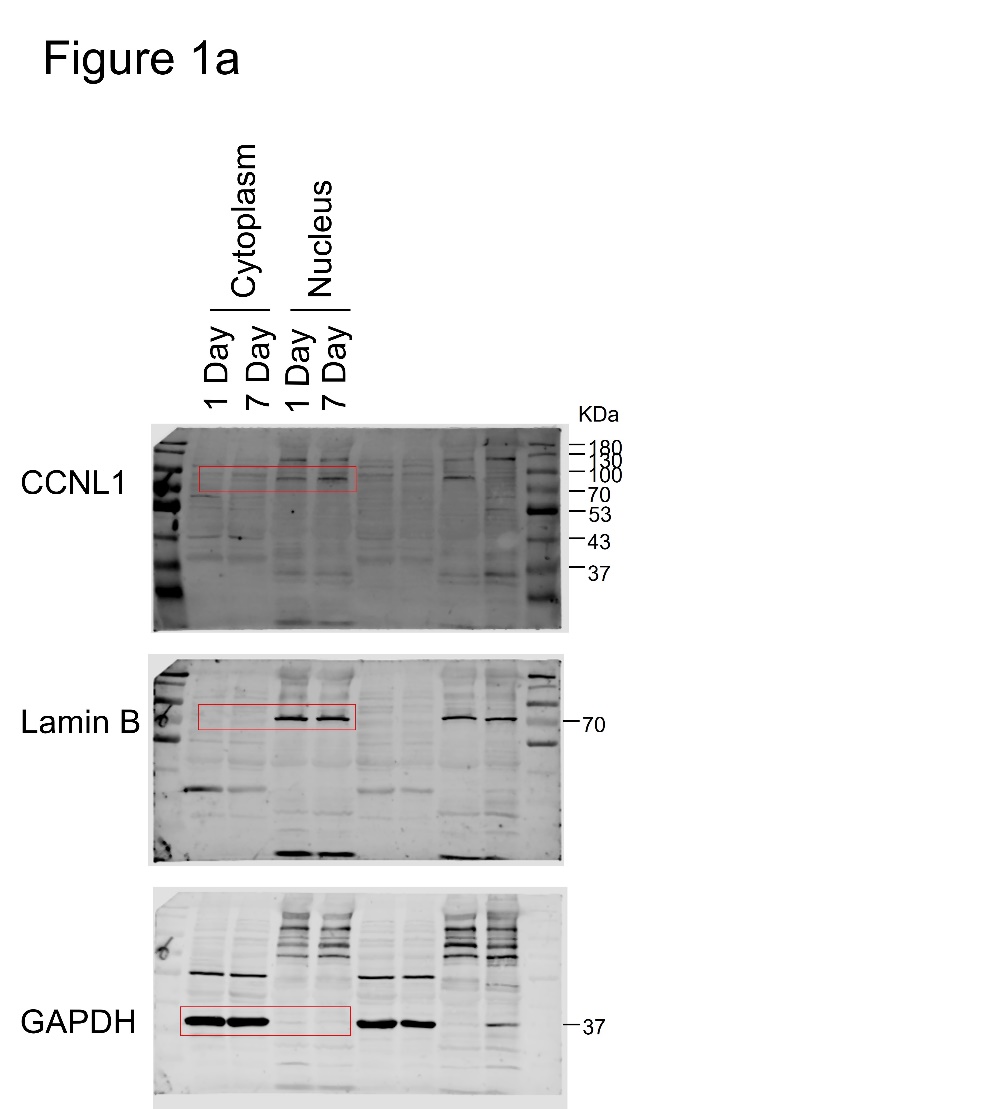

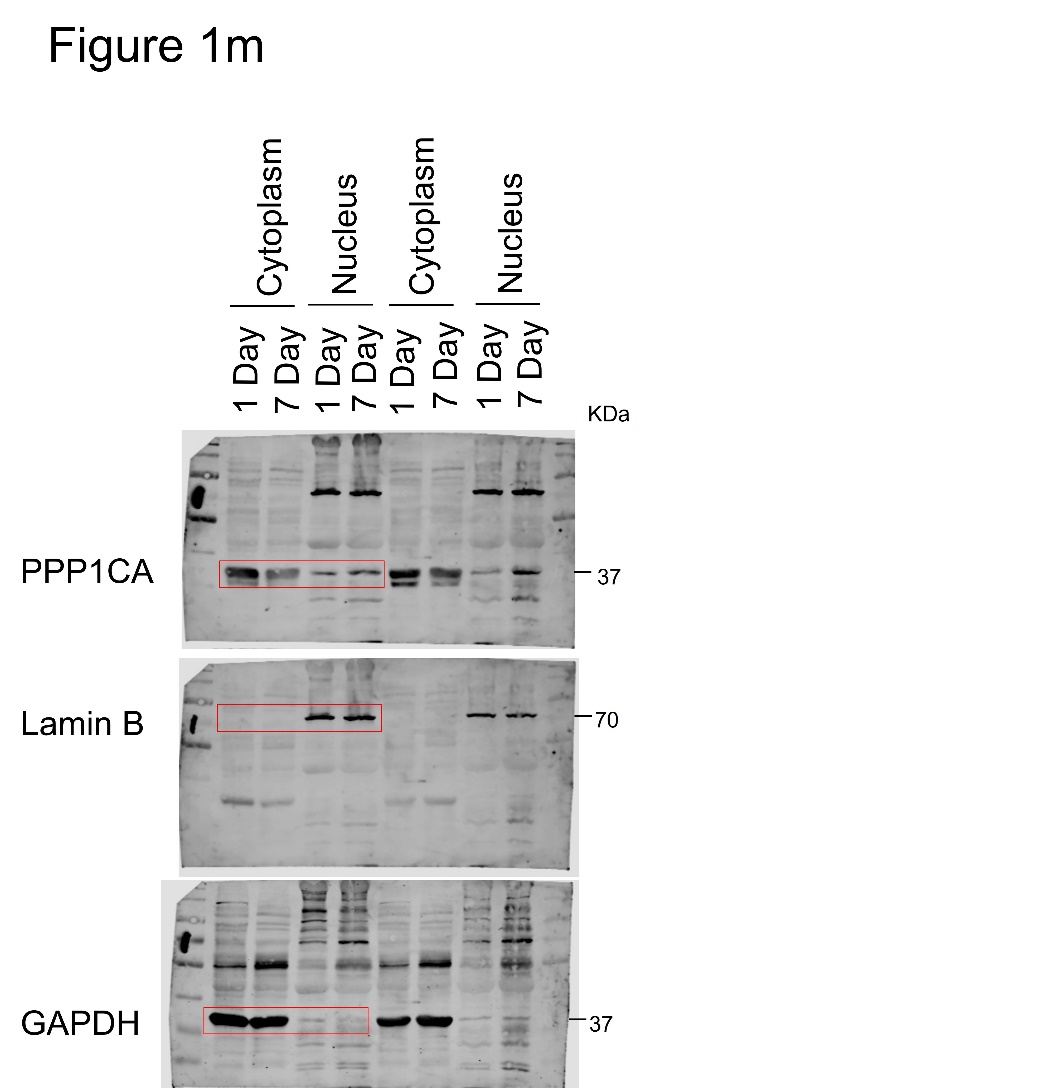
**

**
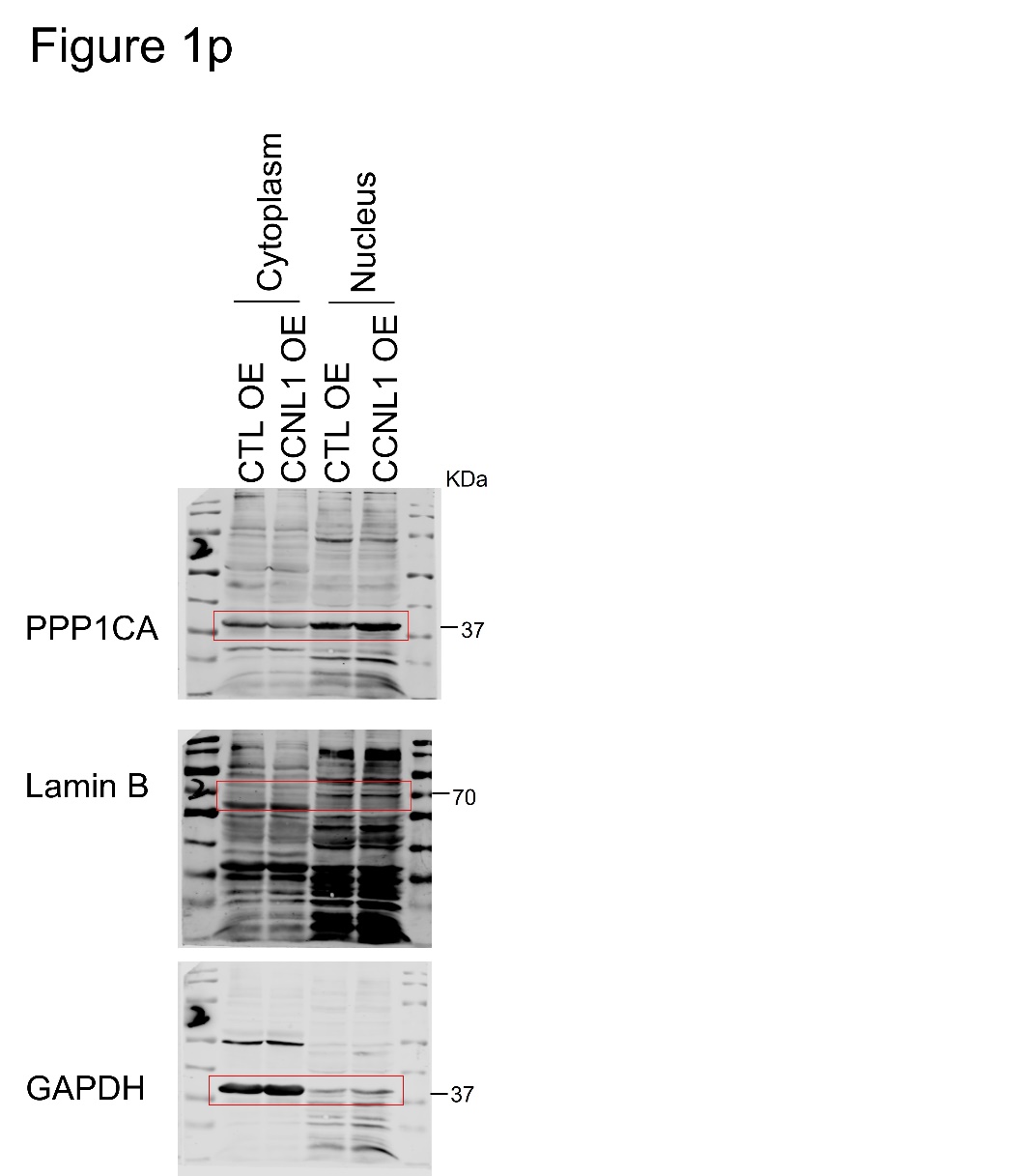

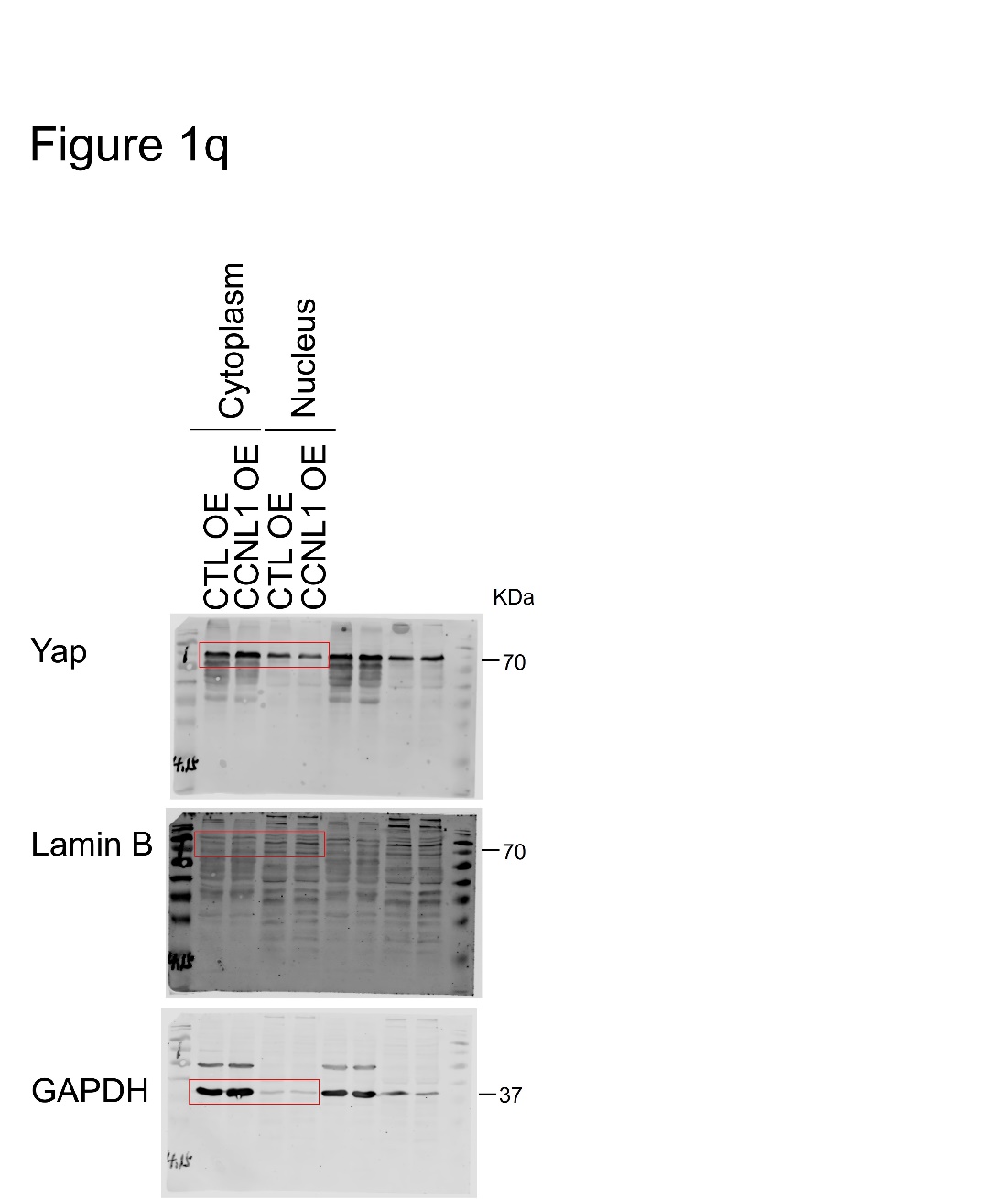
**

**Supplementary Figures:**

**
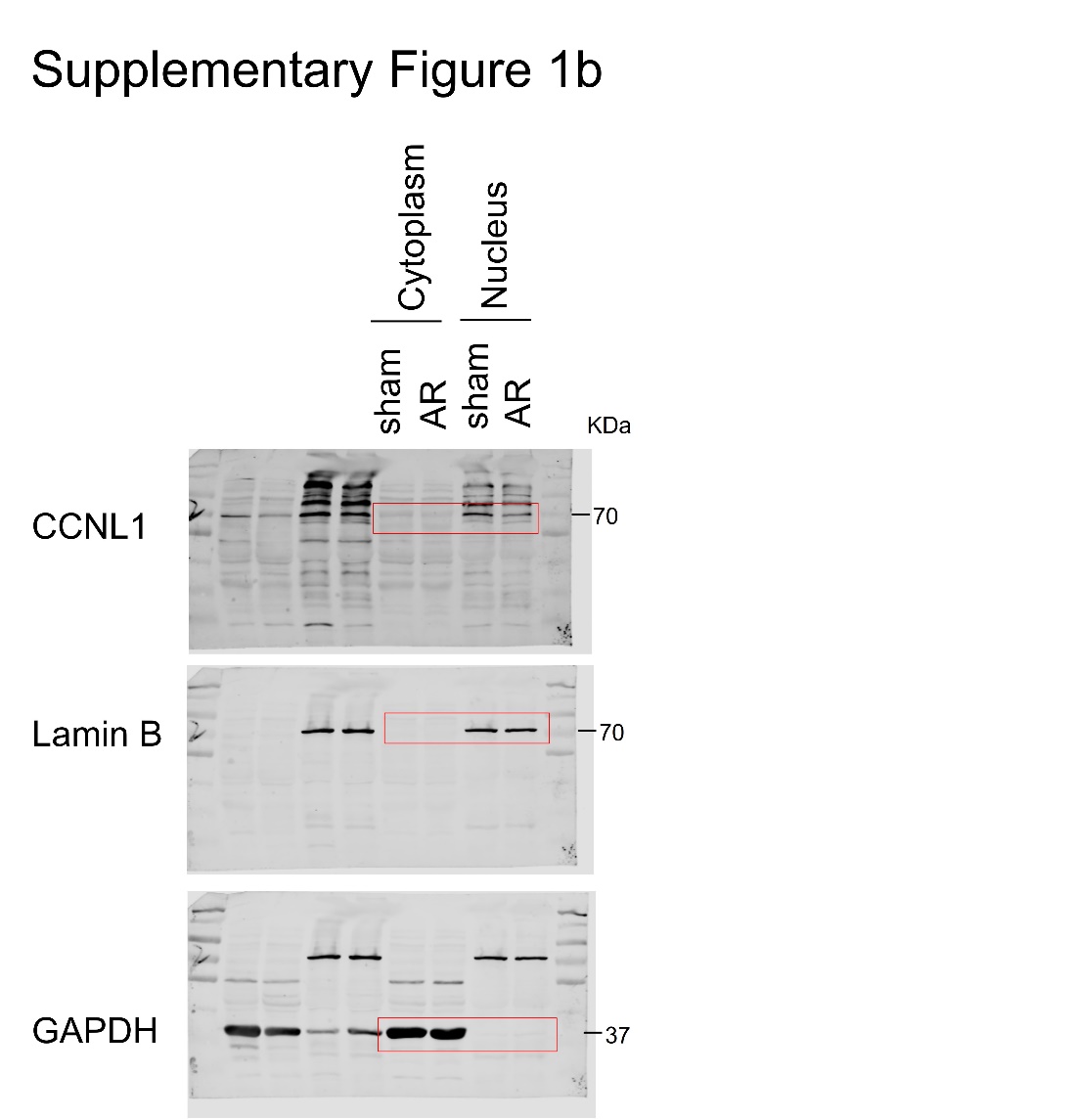

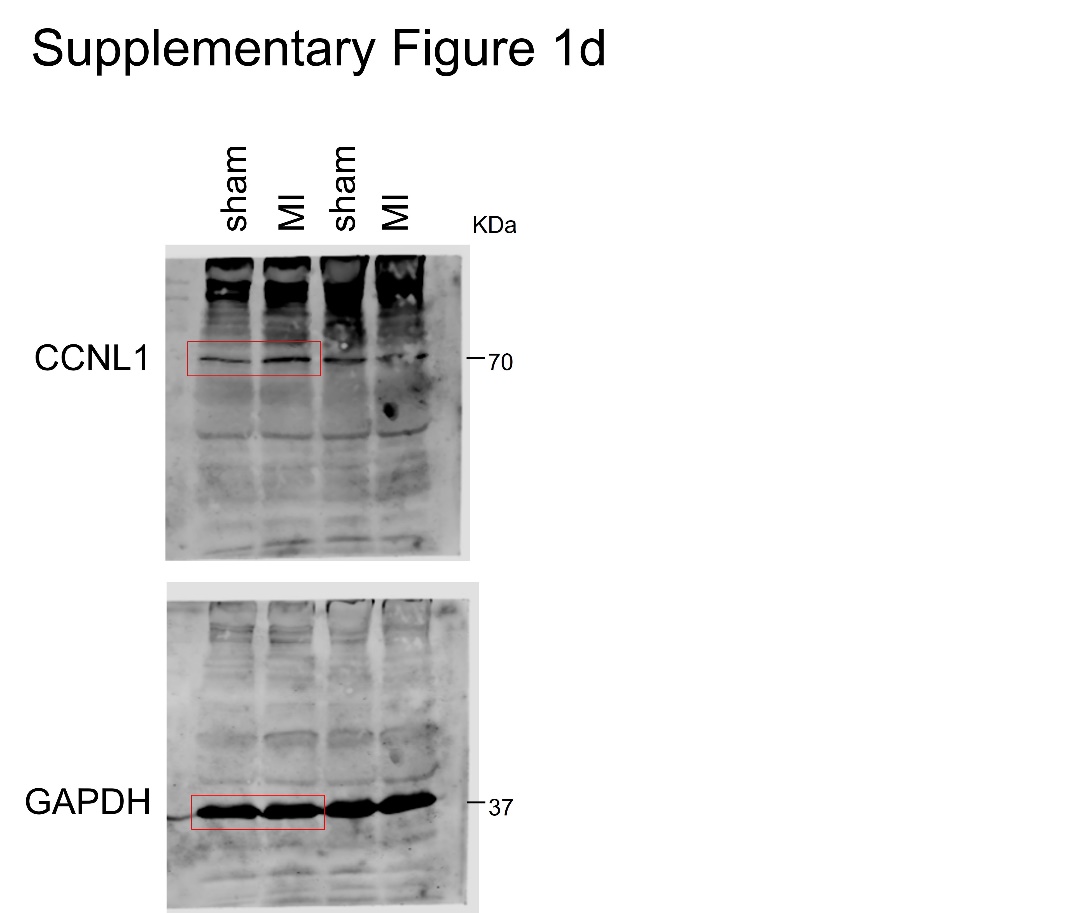
**

**
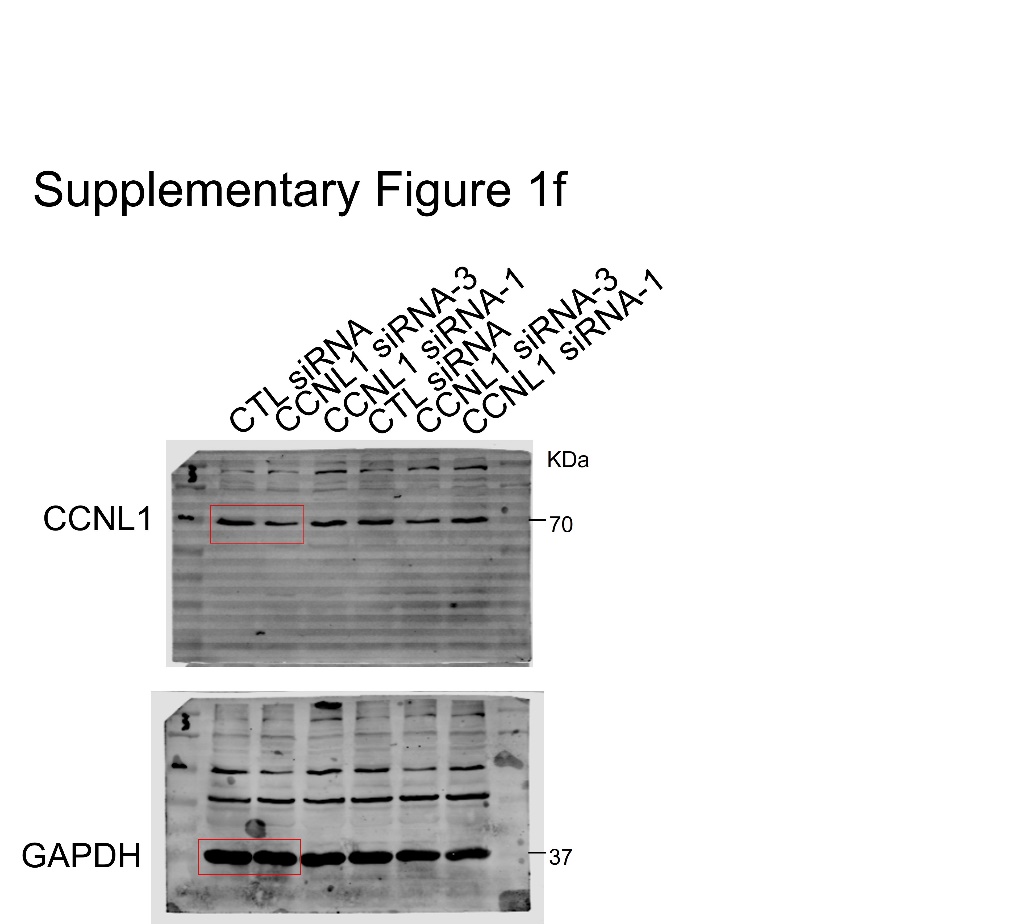

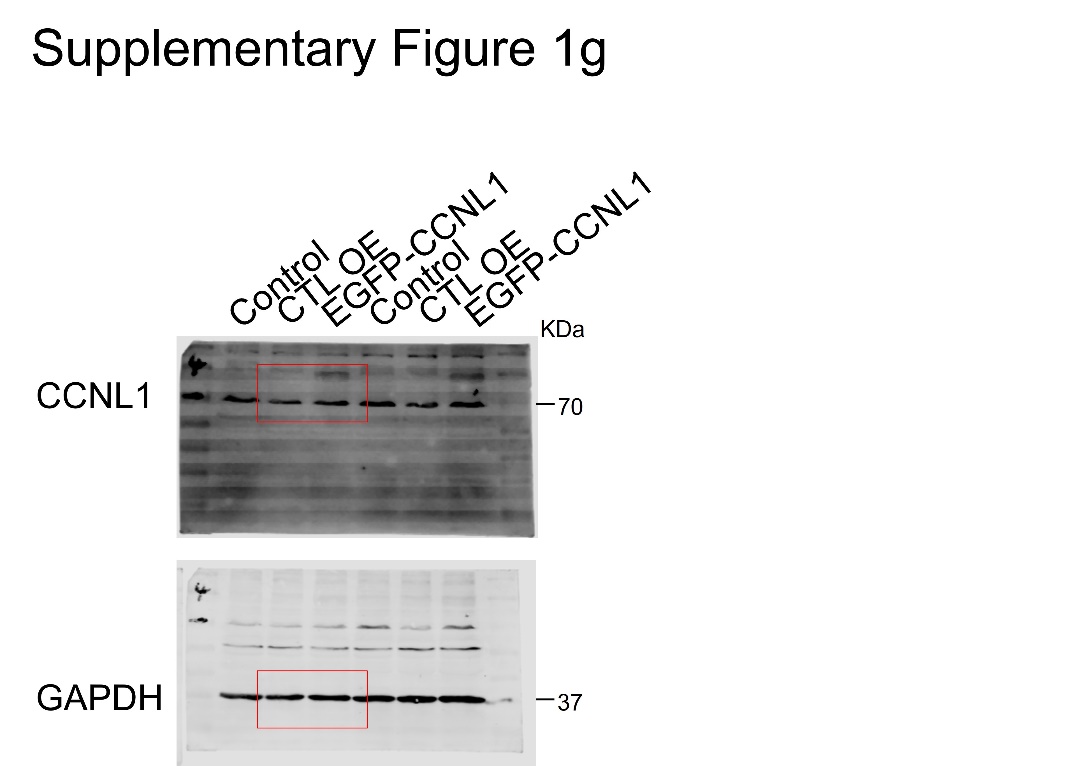
**

**
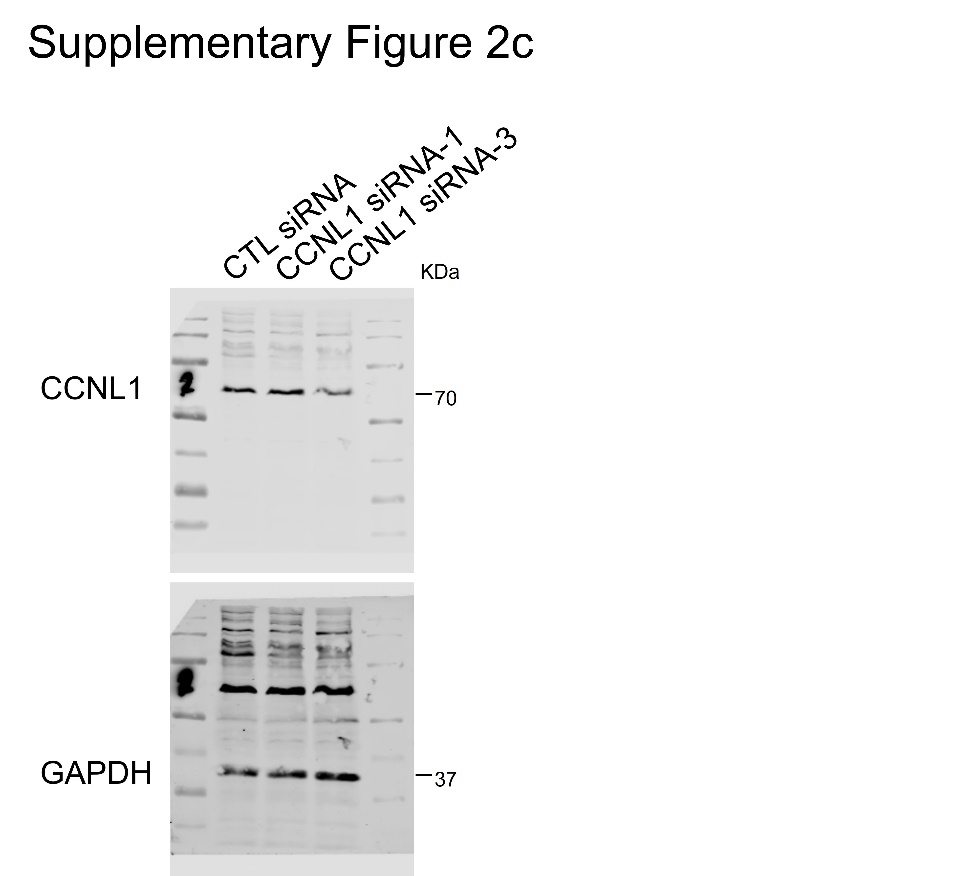

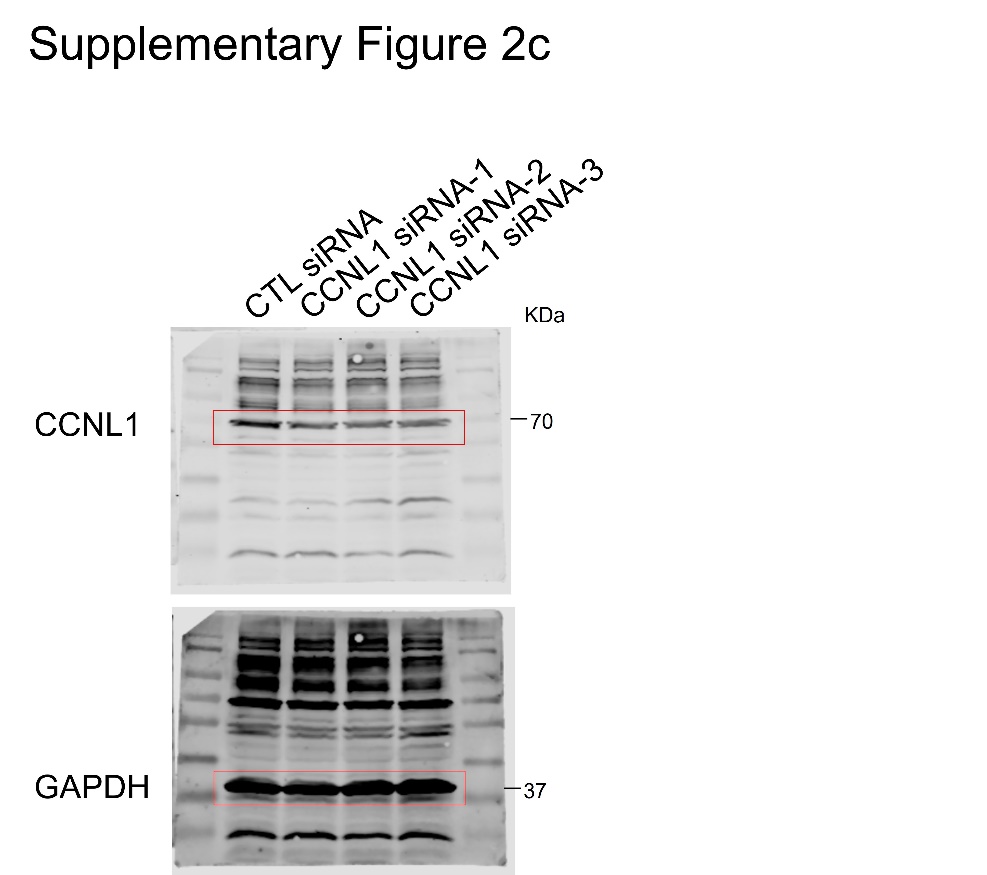
**

**
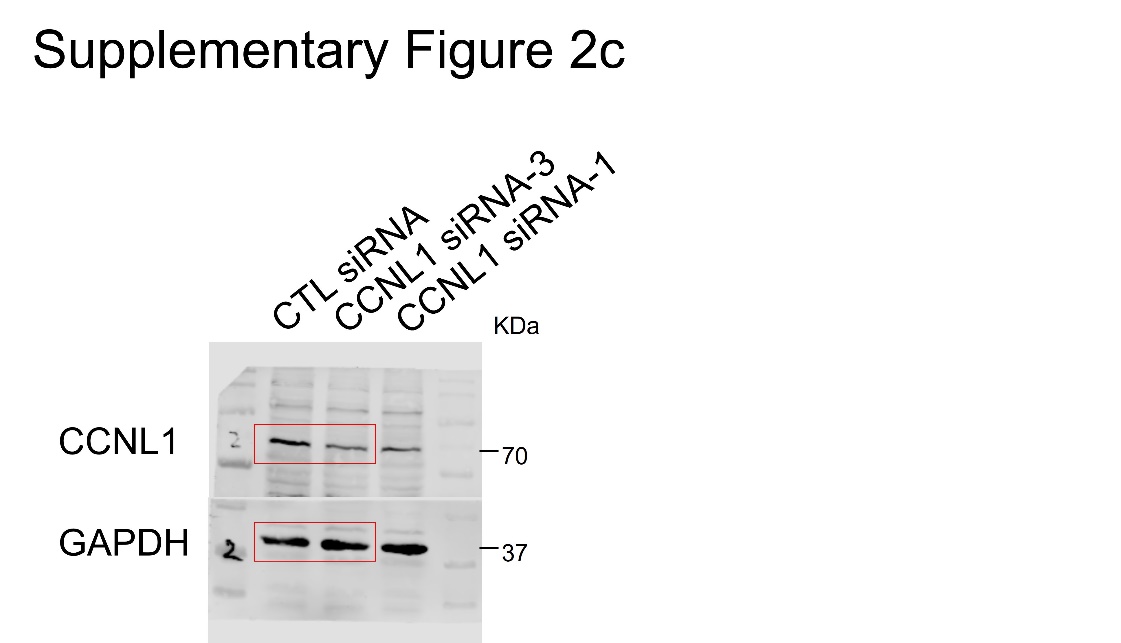

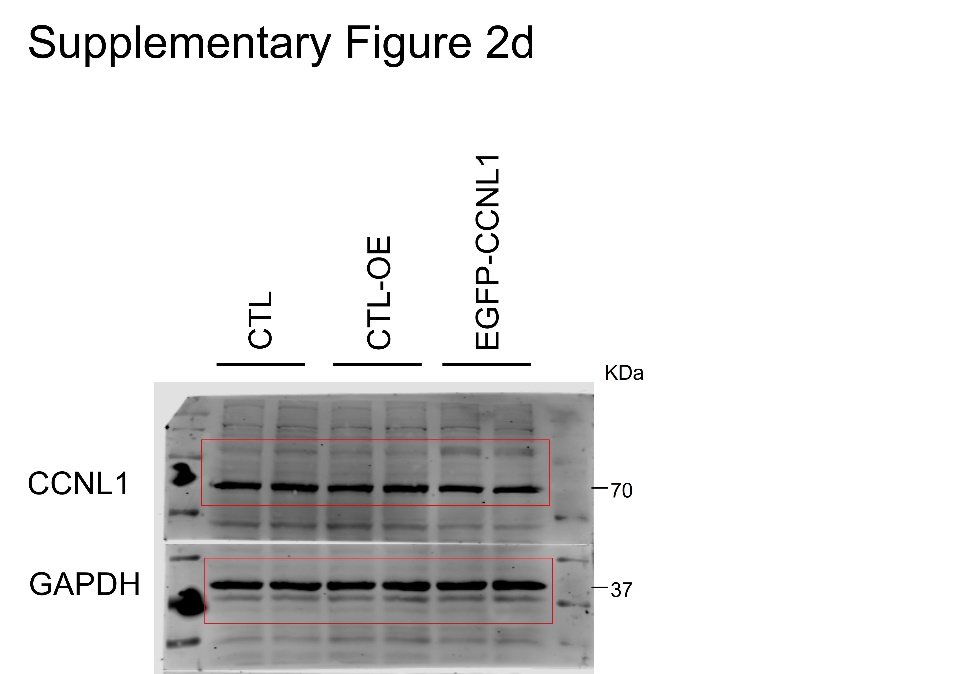
**

**
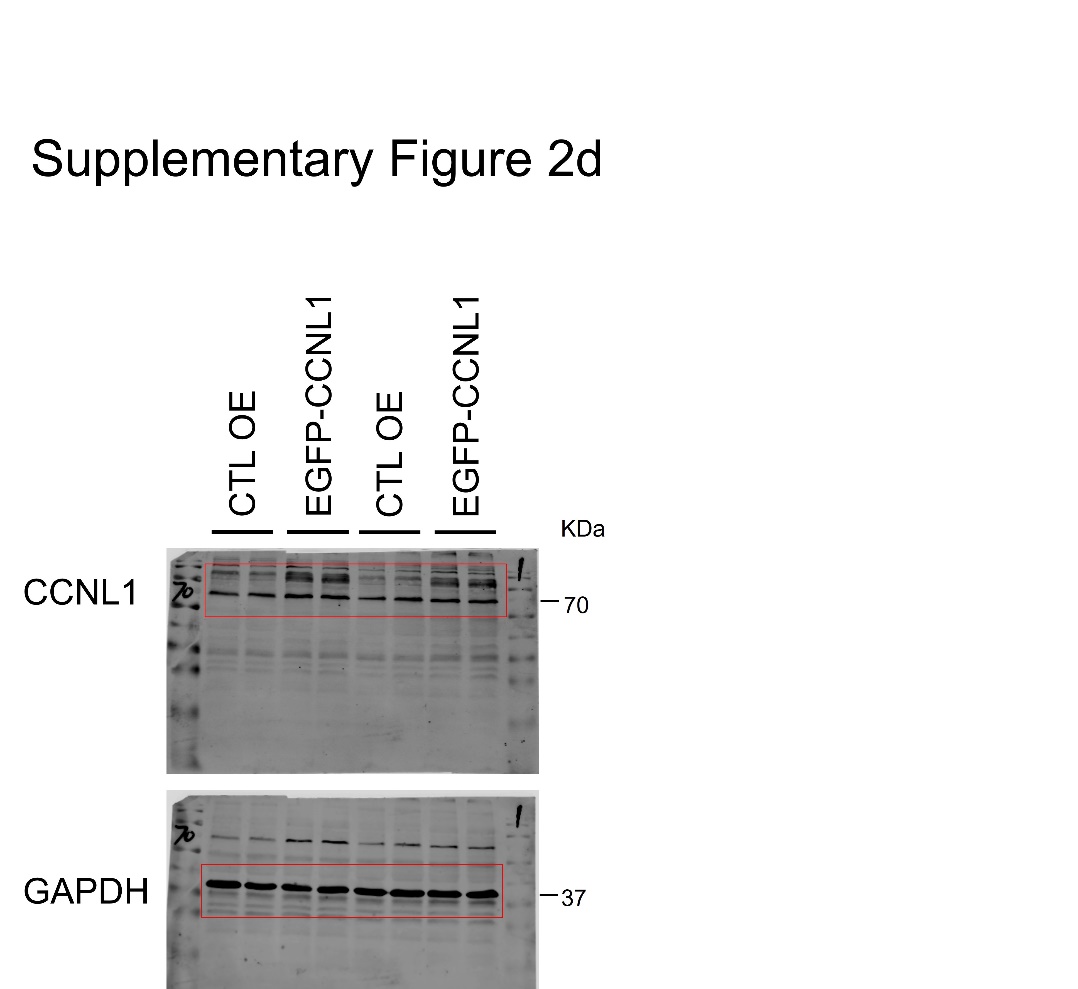

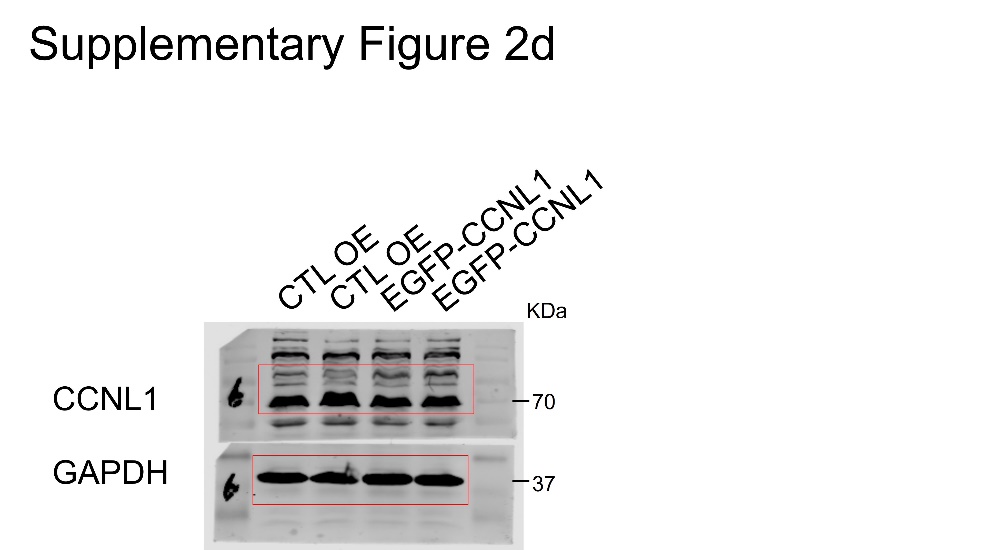
**

**
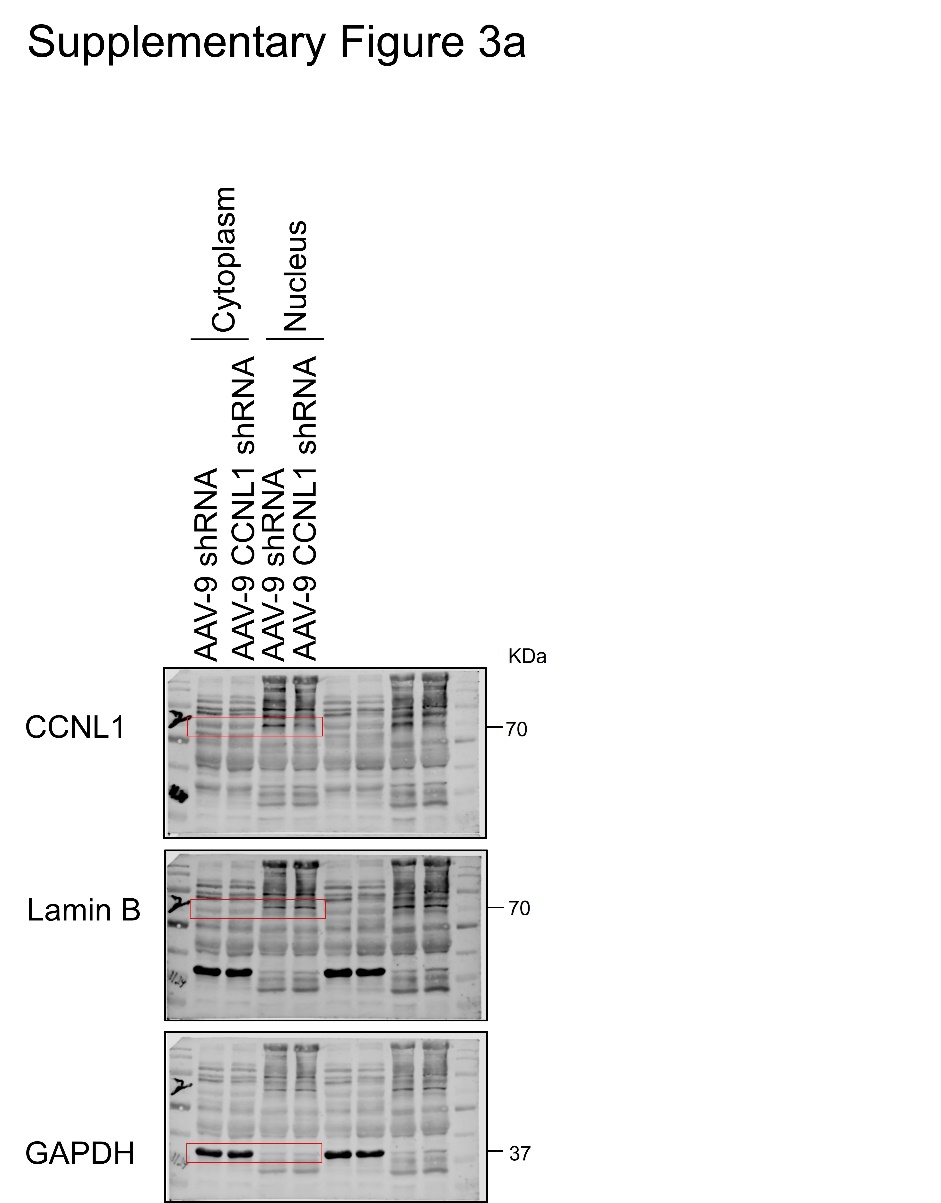

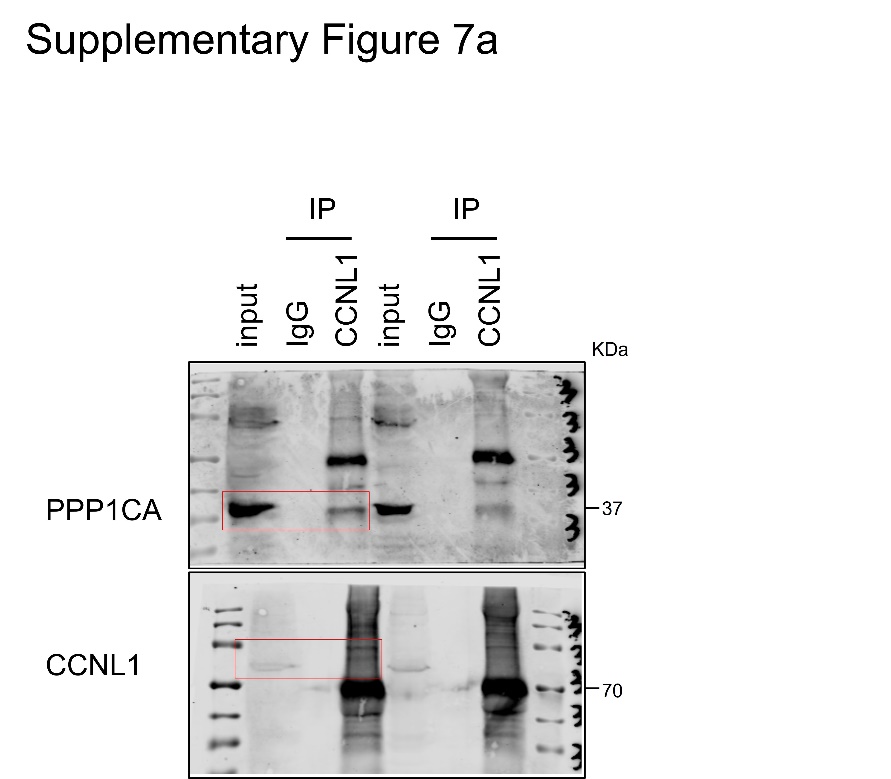
**
